# Triazatruxene: A Rigid Central Donor Unit for a D–A_3_ Thermally Activated Delayed Fluorescence Material Exhibiting Sub‐Microsecond Reverse Intersystem Crossing and Unity Quantum Yield via Multiple Singlet–Triplet State Pairs

**DOI:** 10.1002/advs.201700989

**Published:** 2018-04-16

**Authors:** Paloma L. dos Santos, Jonathan S. Ward, Daniel G. Congrave, Andrei S. Batsanov, Julien Eng, Jessica E. Stacey, Thomas J. Penfold, Andrew P. Monkman, Martin R. Bryce

**Affiliations:** ^1^ Department of Physics Durham University South Road Durham DH1 3LE UK; ^2^ Department of Chemistry Durham University South Road Durham DH1 3LE UK; ^3^ Chemistry School of Natural and Environmental Sciences Newcastle University Newcastle upon Tyne NE1 7RU UK

**Keywords:** fast reverse intersystem crossing rates, thermally activated delayed fluorescence (TADF), triazatruxene

## Abstract

By inverting the common structural motif of thermally activated delayed fluorescence materials to a rigid donor core and multiple peripheral acceptors, reverse intersystem crossing (rISC) rates are demonstrated in an organic material that enables utilization of triplet excited states at faster rates than Ir‐based phosphorescent materials. A combination of the inverted structure and multiple donor–acceptor interactions yields up to 30 vibronically coupled singlet and triplet states within 0.2 eV that are involved in rISC. This gives a significant enhancement to the rISC rate, leading to delayed fluorescence decay times as low as 103.9 ns. This new material also has an emission quantum yield ≈1 and a very small singlet–triplet gap. This work shows that it is possible to achieve both high photoluminescence quantum yield and fast rISC in the same molecule. Green organic light‐emitting diode devices with external quantum efficiency >30% are demonstrated at 76 cd m^−2^.

## 1 Introduction

Organic light‐emitting diodes (OLEDs) have become a central part of materials research, with the ever‐growing requirement for more efficient, higher quality display devices. There is significant interest in OLED materials which emit light via a thermally activated delayed fluorescence (TADF) mechanism^1^ that converts dark, triplet excited states to emissive singlet states by reverse intersystem crossing (rISC). This can be achieved using aromatic donor–acceptor (D–A) molecules, which typically are conjugationally separated with the D and A units orthogonal. These systems emit from a singlet charge transfer state (^1^CT), which is energetically very close to its ^3^CT state through minimized electron exchange. A further excited state such as a local excited triplet state (^3^LE) situated very close in energy to this ^1^CT is also required.^2^ Therefore, having a small singlet–triplet gap (Δ*E*_ST_) is crucial, but is not the only requirement for efficient rISC. rISC can harvest up to 100% of triplet states into singlet states.^3^ Currently, the main challenges facing the TADF community are the long overall residence times of emitter molecules in triplet excited states, and the low oscillator strengths of the ^1^CT radiative transitions. Here, we report a new TADF molecular design, incorporating a rigid, planar, central donor unit with multiple acceptor units bound via C—N bridges. This new design gives a key step forward in TADF efficiency through multiple coupled singlet–triplet states. The resulting fast rISC rates lead to delayed fluorescence (DF) emission lifetimes shorter than the phosphorescence lifetimes of most Ir complexes currently used in OLEDs.^4^ Critically, a unity photoluminescence quantum yield (PLQY) is also maintained.

**Scheme 1:**
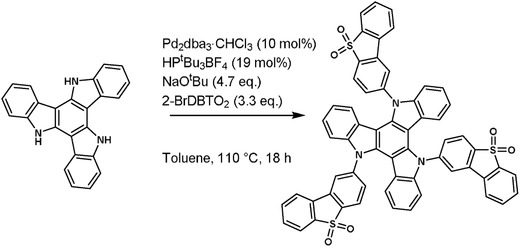
The synthesis of TAT‐3DBTO_2_ via Buchwald–Hartwig coupling conditions.

Recent research has shown that the underlying spin–flip mechanism in rISC is a second order spin–vibronic process.^2^, ^5^ Here, specific molecular vibrations promote mixing between a manifold of singlet and triplet states driving efficient rISC. Other vibrational modes can contribute more to nonradiative decay, requiring careful molecular design.^6^ Gibson and Penfold have recently published further work detailing this process.^7^ Previously synthesized, 1‐substituted phenothiazine (D) D–A–D TADF candidates show molecular restriction with several conformers in solution on the ^1^H NMR timescale.^8^ As well as the phenothiazine donor being tilted, it is clear that there is some rotational restriction around the C—N bond in these systems, which switches off TADF due to a lack of vibronic coupling. The conformation of the phenothiazine with respect to the acceptor is also important in these molecules.^9^ All of the above factors must be taken into account when considering new molecular designs. Another key challenge in the design of TADF molecules is to balance the rates of rISC (and intersystem crossing, ISC)^7^ with the fluorescence quantum yield (*Φ*_F_). Ideally, the desired molecule should have a *Φ*_F_ close to 1 with a short emissive state lifetime. This requires strong coupling of the ^1^CT to the ground state. However, to ensure near degenerate ^1^CT, ^3^CT, and ^3^LE states (which is a requirement for efficient rISC), D–A orthogonality is required. This effectively decouples the ^1^CT states from the ground state. Therefore, either a compromise is required, or nonradiative quenching to the ground state, i.e., internal conversion, must be dramatically curtailed. The new TADF molecule based on a triazatruxene central donor functionalized with three peripheral acceptors, **TAT‐3DBTO_2_** (**Scheme** **1**) is now shown to overcome many of the issues faced when designing an efficient TADF emitter.

## 2 Results and Discussion

### 2.1 Synthesis and Chemical Characterization

The design of **TAT‐3DBTO_2_** (Scheme 1) is based upon the reversal of the donor and acceptor motif typically found in current TADF emitters.^10^ **Tri‐PXZ‐TRZ** was prepared by Tanaka et al. and has a central triazine acceptor unit and three peripheral phenoxazine donor units; this work shows the benefits of symmetry as the **PXZ‐TRZ** and bis‐**PXZ‐TRZ** analogs showed lower device efficiency compared to the C_3_ symmetric **Tri‐PXZ‐TRZ**. In **TAT‐3DBTO_2_** the threefold C_3_ symmetry is maintained, as in **Tri‐PXZ‐TRZ**, but the donor is a central triazatruxene core (**TAT**). Onto this, three dibenzothiophene‐*S*,*S*‐dioxide units are attached via the nitrogen atoms of the core. Dibenzothiophene‐*S*,*S*‐dioxide was selected as the acceptor in an attempt to match the energy of the ^3^LE triplet level of the donor to the ^1^CT energy level. This takes into account that triazatruxene is more electron rich than carbazole due to the central 1,3,5‐trinitrogen‐substituted benzene core, and is also more conjugated, giving a smaller Δ*E*_ST_ and faster rISC rate with dibenzothiophene‐*S*,*S*‐dioxide acceptors. Correctly functionalized **TAT** derivatives can also exhibit high PLQYs.^11^ The synthesis and characterization of **TAT‐3DBTO_2_** is shown in Scheme 1 and Sections S1–S4 (Supporting Information).

**TAT‐3DBTO_2_** was synthesized in 40% yield and has good solubility in various organic solvents, allowing for efficient synthesis and purification of the molecule. This is an efficient synthesis considering that three Buchwald–Hartwig couplings were performed within one overnight reaction.

**Figure 1:**
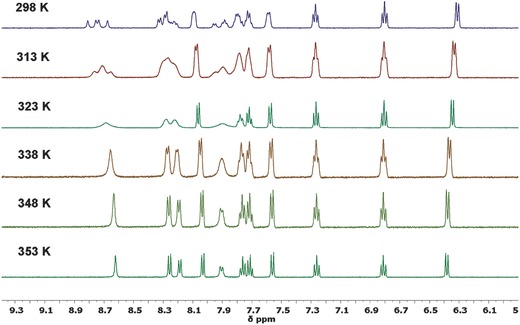
Temperature dependent solution state ^1^H NMR spectra of **TAT‐3DBTO_2_** in dimethylsulfoxide‐d6 (DMSO‐d_6_). The VT NMR data in combination with pure shift ^1^H NMR studies (Figure S6, Supporting Information) shows that there are different conformers of **TAT‐3DBTO_2_** in solution and ^1^H ROESY NMR experiments (Figure S9, Supporting Information) confirm that these conformers all interconvert between each other.

The ^1^H NMR spectrum of **TAT‐3DBTO_2_** at room temperature (298 K) shows a mixture of broad and sharp peaks, suggesting that parts of the molecule are rotating slowly on the NMR timescale, giving rise to multiple environments for the same protons. This has been shown to be the case by using variable temperature (VT) ^1^H NMR, see **Figure** **1**, and further 2D NMR experiments in the Figures S5–S9 (Supporting Information).

The VT NMR data in combination with pure shift ^1^H NMR studies (Figure S6, Supporting Information) show that there are different conformers of **TAT‐3DBTO_2_** in solution; and ^1^H rotating frame nuclear Overhauser spectroscopy (ROESY) NMR experiments (Figure S9, Supporting Information) confirm that these conformers all interconvert between each other. The VT ^1^H NMR data indicate that there is an energy barrier to rotation around the D–A bridging bond and that increasing the temperature to 353 K overcomes this barrier. It is suggested that these conformers relate to the orientation of the three acceptor units with respect to each other and the triazatruxene core (see Section 2.2).

The highest occupied molecular orbital (HOMO) and lowest unoccupied molecular orbital (LUMO) energies for **TAT‐3DBTO_2_** were estimated by cyclic voltammetry and differential pulse voltammetry (see Figure S10 in the Supporting Information) at −5.60 and −3.00 eV, respectively. Within the respective solvent windows, as well as a reversible single‐electron reduction, **TAT‐3DBTO_2_** displays three reversible well‐resolved single‐electron oxidations (Δ*E*_1/2_ *E* ^ox(1)^/*E* ^ox(2)^ = 487 mV, Δ*E*_1/2_ *E* ^ox(2)^/*E* ^ox(3)^ = 603 mV). These correspond to sequential oxidations of the **TAT** core unit.

**Figure 2:**
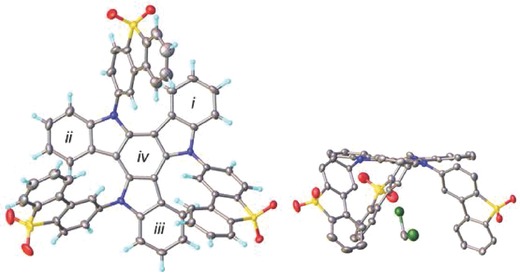
X‐ray crystal structure of **TAT‐3DBTO_2_**. Two views (perpendicular and side‐on to the donor core) are shown. Displacement ellipsoids are drawn at the 50% probability level. CH_2_Cl_2_ solvent molecules are trapped in molecular clefts/cavities or disordered in intermolecular voids of the host.

Crystallization of **TAT‐3DBTO_2_** was challenging, although single crystals suitable for X‐ray analysis were obtained (**Figure** **2**). It is suggested that crystal packing (see Section S11 in the Supporting Information) is responsible for the obtained structure having all acceptor units on the same face of the donor plane. In coevaporated amorphous films, many different conformations of the molecule should coexist. The ground state D–A dihedral angles range from 58° to 62°, significantly less that 90°, which is very much in line with one of the current most efficient TADF materials for green OLEDs, 1,2,3,5‐tetrakis(carbazol‐9‐yl)‐4,6‐dicyanobenzene (4CzIPN).^12^

### 2.2 Quantum Chemistry Studies

**Figure 3:**
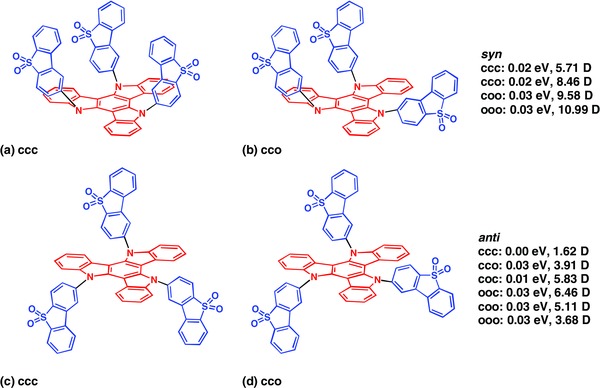
Different possible conformations of the **TAT‐3DBTO_2_** molecule. The stable conformations of the donor (red) and three acceptor units (blue) found by DFT(PBE0) structural investigations, in line with the measured solution state NMR spectra. *Syn* is for conformations with all the acceptors pointing in the same direction, and *anti* represents conformations for which one acceptor points in a different direction. c = closed, o = open as illustrated in a) ccc, b) cco, c) ccc, and d) cco. This naming convention is explained in Section S12 (Supporting Information).

**Figure** **3** shows the ten possible conformers of **TAT‐3DBTO_2_**, all of which are within 0.03 eV of each other, reflecting the results from the VT ^1^H NMR studies (Figure 1). The different conformations consist of different combinations of acceptor orientations with respect to the donor moiety.

#### 2.2.1 Electronic Structure—Absorption

**Figure 4:**
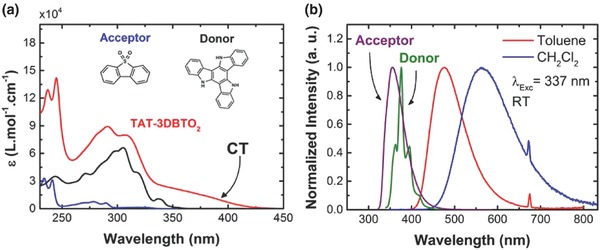
Absorption and emission characteristics of **TAT‐3DBTO_2_** in solution. a) Extinction coefficient spectra of the acceptor (A), donor (D), and **TAT‐3DBTO_2_** molecules, all diluted in dichloromethane (CH_2_Cl_2_) solvent. Inset graph also shows the chemical structure of A and D units. b) Normalized photoluminescence (PL) spectra of acceptor (data taken from ref. 20), donor, and **TAT‐3DBTO_2_** molecules. D and A are diluted in toluene solvent, **TAT‐3DBTO_2_** is diluted in toluene and CH_2_Cl_2_. The peak around 674 nm is the second order peak from the excitation beam.

The absorption spectrum of **TAT‐3DBTO_2_** has been computed in the gas phase including the twenty lowest singlet and triplet states. The absorption spectrum exhibits three dominant peaks (see Section S13 in the Supporting Information) at 410, 350, and 290 nm. The intensity of each peak increases with higher energy and there is reasonable agreement with the experimental spectrum shown in **Figure** **4**. The molecular conformation has negligible effect on the position, intensity, or character of the transitions and all of the excited states computed exhibit charge transfer character from the donor core to the acceptor moieties, as shown by the density difference plots for each state shown in Section S13 (Supporting Information). The first absorption peak is composed of six pairs of singlet and triplet states of the same character all lying within 0.3 eV of each other. The difference densities associated with each transition are shown in Section S13 (Supporting Information). Because of the C_3_ symmetry at this geometry, the states T_2_ and T_3_ (S_2_ and S_3_) as well as states T_5_ and T_6_ (S_5_ and S_6_) are degenerate. At the ground state equilibrium geometry, the molecular dipole moment is found to be between 1 and 11 D depending on the conformer (see Figure 3).

#### 2.2.2 Electronic Structure—Emission/Relaxation

The emission energy for each conformer is simulated by optimizing the lowest singlet and triplet states. In each case, a rotation of one acceptor moiety to become nearly orthogonal with respect to the donor core is observed. This motion allows minimization of the overlap between the orbitals involved in the excitation and therefore decreases the singlet–triplet energy gap (∆*E* ^T1/S1^ = 0.01 eV). The minimum energy geometry of both S_1_ and T_1_ is very similar as both states are pure charge transfer states from the donor core to one of the acceptor moieties (see Section S14 in the Supporting Information). The Stokes shift of the S_1_ and T_1_ states (difference between the energy at ground state and excited state optimized geometries) is 0.46 and 0.29 eV, respectively. This Stokes shift difference is because the states change character slightly between the Franck–Condon (ground state)–optimized geometry and the S_1_/T_1_ optimized geometry. For the latter, they are pure CT states from HOMO (donor) to LUMO on one acceptor, but for the former, they are donor to an orbital delocalized over all the acceptors. Also, there is more orbital overlap between the donor and acceptors at the ground state geometry because they are not orthogonal. Therefore, there is a bigger S_1_–T_1_ gap, which is why the Stokes shift is larger for S_1_ than T_1_. The calculations clearly indicate that in the excited state geometry, the structure changes such that the singlet relaxes more than the triplet because the new structure minimizes the exchange energy. Thus, in the excited state geometry, the S_1_ and T_1_ states are of equal energy as required for optimal TADF.

**TAT‐3DBTO_2_** has three acceptors, which are quasi equivalent. Consequently, one can expect three energy minima in S_1_ corresponding to a charge transfer from the donor core to each of the different acceptor moieties. We thus find that within 0.2 eV of the T_1_ state, there are 12 excited states, all of which are likely to be vibrationally coupled. If one considers all of the angular momentum components (i.e., all M_s_ levels of the triplets), then **TAT‐3DBTO_2_** potentially has 30 coupled states involved in rISC. This will give significant enhancement to the rISC rate.

The oscillator strength of **TAT‐3DBTO_2_** is very similar (0.001) to a literature TADF material **DPTZ‐DBTO_2_** 3 (0.0007), as expected by the similar lifetime of the prompt emission in both molecules. Consequently, from these quantum chemistry simulations, it would appear that the enhanced performance of **TAT‐3DBTO_2_** is primarily associated with the enhanced rISC rate derived from the higher density of states.

### 2.3 Photophysical Properties

#### 2.3.1 Solution Properties

Figure 4a shows the extinction coefficient spectra of **TAT‐3DBTO_2_** and the individual D and A units in dichloromethane (CH_2_Cl_2_). By comparison to the individual D and A units, the extinction coefficient at all wavelengths is greatly enhanced in **TAT‐3DBTO_2_**. This increase in absorption intensity strongly reflects the higher density of states predicted from the quantum chemical calculations described above. Particularly, the absorption band at lower energy (350–425 nm), which is not observed in the D or A units, and is ascribed to a direct absorption from the CT states,^3^ is very strong in this new material. Figure S15 (Supporting Information) shows a slight redshift on the right edge of the spectra by increasing the polarity of the solvent, which is associated with a strongly mixed n → π* /π → π* character transition,^13^ also confirmed by its relatively strong transition. Excitation into this band directly populates ^1^CT excited states, as we have shown in the analogous D–A–D system **DPTZ‐DBTO_2_**.^3^

Figure 4b shows the photoluminescence (PL) spectra of **TAT‐3DBTO_2_** in different solvents together with the separated D and A units in toluene solution. The spectra show clear and strong CT emission, displaying a Gaussian band shape and strong redshift compared to the individual D and A emission spectra. The PL spectra shift to longer wavelengths upon increasing the solvent polarity. This indicates strong positive solvatochromism, as observed in other D–A–D‐type molecules.[[qv: 1f,14]]

#### 2.3.2 Solid State Properties

BCPO, (*bis*‐4‐(*N*‐carbazolyl)phenyl)phenylphosphine oxide)^15^ was used as a host for **TAT‐3DBTO_2_** to maintain the low energy splitting between ^1^CT and ^3^LE with correct host polarity. The polarity of the P=O bond in BCPO redshifts the ^1^CT energy compared to 1,3‐bis(N‐carbazolyl)benzene (mCP) matrix. The ^1^CT is tuned so that it is very close in energy to the ^3^LE state. Tuning the Δ*E*_ST_ in such a fashion has previously been demonstrated in the literature. The correct host polarity in this context is defined as a host with a polarity that will minimize the Δ*E*_ST_.[[qv: 14a,b]] Furthermore, a PLQY of approximately unity was obtained from evaporated films of **TAT‐3DBTO_2_**:BCPO (see Section S16 in the Supporting Information).

**Figure 5:**
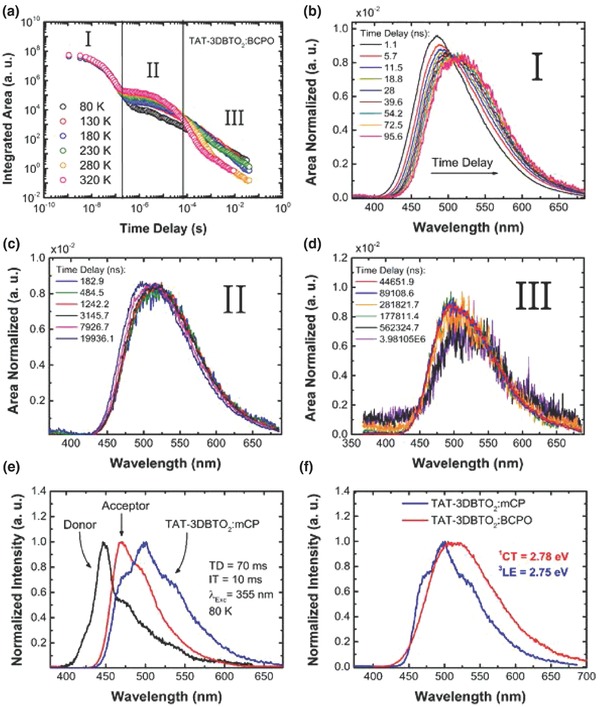
Time‐resolved fluorescence decay of **TAT‐3DBTO_2_**:BCPO and **TAT‐3DBTO_2_**:mCP films. a) Intensity decay from nanosecond to millisecond of **TAT‐3DBTO_2_**:BCPO film at different temperatures. b–d) Time‐resolved area normalized emission spectra in regions I, II, and III, respectively. e) Normalized phosphorescence (PH) spectra of acceptor (data taken from ref. 20), donor, and **TAT‐3DBTO_2_**:mCP, all collected at 80 K. f) Normalized photoluminescence (PL) spectra of **TAT‐3DBTO_2_**:BCPO and PH of **TAT‐3DBTO_2_**:mCP. All spectra were obtained with 355 nm excitation. Saturation of the DF above 280 K is shown in Section S17 (Supporting Information).

Three different emission decay regimes are observed, **Figure** **5**a: region I is fast decay, associated with prompt CT emission (PF); region II is early time DF; and region III is long‐lived DF. The PF decay curves show no temperature dependence, indicating negligible migration of the singlet ^1^CT excited state. The decay curve at 320 K was fitted using a biexponential function: τ_1_ = 10 ns (*I*_1_ = 4.4) and τ_2_ = 35 ns (*I*_2_ = 1.2). Consequently, τ_average_ = 22.4 ns for region I (see Section S17 in the Supporting Information). Region II shows strong TADF, the DF emission increasing in intensity with increasing temperature. The decay times related to region II are τ_1_ = 103.9 ns (*I*_1_ = 42 777), τ_2_ = 3.2 µs (*I*_2_ = 112 006), and τ_3_ = 15.1 µs (*I*_3_ = 61 040). Consequently τ_average_ = 11.7 µs for region II (see Section S17 in the Supporting Information). Usually, DF lifetimes of TADF emitters are in the microsecond timeframe, whereas **TAT‐3DBTO_2_** has a DF component with a lifetime on the order of 100 ns, which is a result of fast rISC. Hofbeck and Yersin showed that *fac*‐Ir(ppy)_3_ has multiple component emission lifetimes (τ_1_ = 200 ns, τ_2_ = 6.4 µs, τ_3_ = 116 µs).^4^ The fastest delayed component of **TAT‐3DBTO_2_** emission (103.9 ns) is faster than any emission component from *fac*‐Ir(ppy)_3_. This demonstrates that fast emission can be achieved without the need of a heavy metal.

As the PLQY for **TAT‐3DBTO_2_** in BCPO is ≈1, this is not a quenched component, and from its fit weighting it represents ≈20% of all the delayed emissions. This complex multicomponent DF decay is ascribed directly to the multiple conformations possible in **TAT‐3DBTO_2_**. Region III has an inverse temperature dependence: the intensity of the emission increases as the system temperature drops. This has been observed before in highly efficient TADF molecules, and is associated with longer‐lived DF components and phosphorescence (PH) at low temperatures.[[qv: 14a]]

The reverse intersystem crossing rates (*k*_rISC_) of **TAT‐3DBTO_2_**:BCPO film (320 K) were calculated using two different approaches (see Section S18 in the Supporting Information). Three different values of *k*_rISC_ were calculated (see **Table** **1**), each value is associated with a distinct lifetime of DF in a **TAT‐3DBTO_2_**:BCPO film. The fastest lifetime of the DF emission (τ_1_ in region II) gives very high *k*_rISC_ > 10^7^ s^−1^. Both the *k*_rISC_ calculation methods show good agreement. Calculating the ISC rate, *K*_ISC_, using *Φ*_ISC_ = τ_prompt_·*k*_ISC_ (using the data from Section S18 in the Supporting Information) yields *k*_ISC_ = 3.5 × 10^7^ s^−1^. Taking the fastest *k*_rISC_ = 1.5 × 10^7^ s^−1^ (see Section S18 in the Supporting Information), we see that *K*_rISC_ ≈ *K*_ISC_ and so it is not surprising that the TADF becomes so efficient. The recycling rate of singlet to triplet and back approaches 1 in this case, indicative of very fast and efficient rISC.[[qv: 14c]] Figure 5b shows the area‐normalized emission spectra in region I at 320 K. The PF emission shows a continuous dynamic redshift. This redshift is associated with the energetic relaxation of the ^1^CT state, primarily due to rotation about the D–A bond. Calculations and experiments suggest the D and A units twist toward a more orthogonal geometry, and stabilize in around 70 ns. Region II (Figure 5c) shows stabilized ^1^CT emission at 320 K: the onset of each spectrum collected in this region is at 2.78 ± 0.02 eV. The intensity dependence of the DF emission in this region as a function of the laser excitation dose was found to be linear with a gradient of 1, indicative of TADF (see Section S19 in the Supporting Information). Figure 5d shows late time decay (weak emission). Between 70 and 400 µs (still exponential decay), emission as in region II is observed. From 400 µs to 10 ms (power law decay), very weak emission is detected. Likely, this region includes DF emission from additional conformers and weak PH emission.

**Table 1:** rISC rates determined from the three exponential decays from **TAT‐3DBTO_2_**:BCPO film using approach a $\left(k_{\mathrm{rISC}}^{a}\right)$ and approach b $\left(k_{\mathrm{rISC}}^{b}\right)$ for comparison[[qv: 14a,b]]

Decay component	$k_{\mathrm{rISC}}^{a}=\frac{1}{\tau_{\mathrm{DF}}}\cdot \frac{1}{\left(1-\Phi_{\mathrm{ISC}}\right)}$	$k_{\mathrm{rISC}}^{b}=\;\frac{\int I_{\mathrm{DF}}(t)dt}{\int I_{\mathrm{PF}}(t)dt}\cdot \frac{1}{\tau_{\mathrm{DF}}}$
τ_1_ = 103.9 ns	1.5 × 10^7^ s^−1^	1.3 × 10^7^ s^−1^
τ_2_ = 3.2 µs	4.9 × 10^5^ s^−1^	4.3 × 10^5^ s^−1^
τ_3_ = 15.1 µs	1.0 × 10^5^ s^−1^	9.2 × 10^4^ s^−1^

Spectral analysis at 80 K was performed to identify the PH emission. The harvesting of triplet states to singlet states in BCPO host is so rapid that obtaining a clear PH spectrum is problematic. This is due to residual ^1^CT emission masking the very weak PH emission. Therefore, the PH spectrum was measured in mCP. In this host the ∆*E*_ST_ is larger (0.21 ± 0.03 eV), allowing the PH spectrum to be clearly identified at low temperature (80 K) (see Section S20 in the Supporting Information). The **TAT‐3DBTO_2_**:mCP PH spectrum was also compared to the PH spectrum collected in polyethylene oxide matrix, and both the spectra show the same onset energy (see Section S20 in the Supporting Information).

Figure 5e shows the PH spectra of **TAT‐3DBTO_2_**:mCP film and the A and D units. The PH spectrum of **TAT‐3DBTO_2_** shows mostly ^3^LE character from the acceptor units, while a peak around 550 nm is strongly enhanced. Comparison of the phosphorescence vibronic intensities may indicate a perturbed geometry for the LE triplet state in **TAT‐3DBTO_2_** compared to the isolated acceptor unit. Considering that the ^3^LE states are almost unaffected by the polarity of the host environment, the triplet levels of **TAT‐3DBTO_2_** doped into BCPO will have onsets very close to those observed in mCP and polyethylene oxide.

Figure 5f shows the PH spectrum together with the PL spectrum of **TAT‐3DBTO_2_**:BCPO film, for better comparison. The ^1^CT and ^3^LE states have onset energies of 2.78 ± 0.02 and 2.75 ± 0.02 eV, respectively, leading to ∆*E*_ST_ = 0.03 ± 0.03 eV. Therefore, it is clear that for **TAT‐3DBTO_2_** in BCPO host, the ^1^CT state energy lies very close to the triplet states, as required for fast rISC and highly efficient TADF. However, we also note that there are twelve states very close in energy (described above), which would also couple to mediate rISC.

### 2.4 OLED Performance

BCPO is an ideal ambipolar host for **TAT‐3DBTO_2_**, yielding the minimal Δ*E*_ST_ and a PLQY of ≈100%. Optimization studies concerned finding the best guest:host (*x*:*y*) ratios. Therefore, two different device architectures were used: one designed for optimization of maximum external quantum efficiency (EQE) values (OLED 1) and the another aiming for low roll‐off (OLED 2). For optimization of maximum EQE, a lower amount of **TAT‐3DBTO_2_** was coevaporated with BCPO host, 1:9 v/v, and for optimization of roll‐off the ratio of **TAT‐3DBTO_2_** to host was higher (1.7:8.3). The architecture of the optimized devices was: indium‐tin‐oxide (ITO)/NPB(40 nm)/TCTA(10 nm)/**TAT‐3DBTO_2_**:BCPO(*x*:*y*,30 nm)/TPBi(40 nm)/LiF(1 nm)/Al 100 nm). NPB (*N,N′*‐bis(naphthalen‐1‐yl)‐*N,N′*‐bis(phenyl)‐benzidine) and TCTA (tris(4‐carbazol‐9‐ylphenyl)amine)) were used as commercial hole transport layers, TPBi (1,3,5‐tris(*N*‐phenylbenzimidazol‐2‐yl)benzene) as electron transport layer, LiF (lithium fluoride) as electron injection layer, and Al (aluminum) was used as cathode.

**Figure 6:**
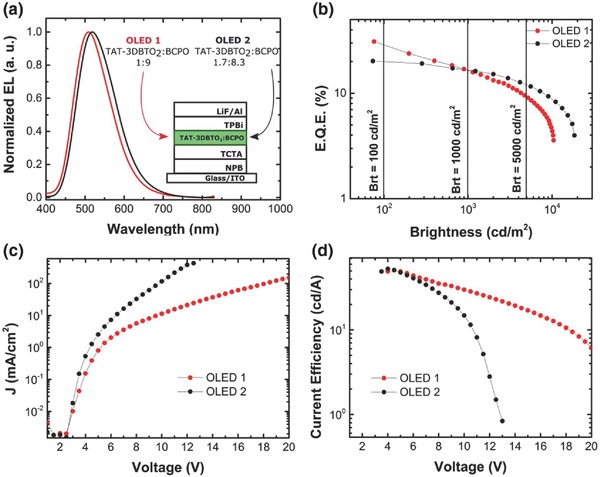
**TAT‐3DBTO_2_** OLED device performance characteristics. a) EL spectra of OLED 1 and OLED 2 and a schematic of the OLED architectures. b) Measured external quantum efficiencies as a function of brightness. c) Current–voltage curves and d) current efficiency as a function of drive voltage, all graphs for both the structures, OLED 1 and OLED 2.

**Figure** **6**a shows the green electroluminescence (EL) spectra of both the devices collected at 10 V. The Commission Internationale de L′Éclairage*_xy_* chromaticity coordinates for these EL spectra are (0.26, 0.46) and (0.29, 0.50) for OLED 1 and OLED 2, respectively. The emission from OLED 2 is slightly redshifted, which is likely associated with the increase in the overall polarity of the emissive layer induced by the increased **TAT‐3DBTO_2_** concentration.

Figure 6b shows representative EQE versus brightness curves. OLED 1 shows a maximum EQE value of 30.9% (76 cd m^−2^). Given this high EQE value, and the fact that the PLQY was found to be ≈100%, it can be concluded that the device has a charge balance (γ) close to unity. This implies that all the triplet excitons are harvested to singlet states, (η_ST_ = 1), assuming an outcoupling efficiency (η_out_) of ≈0.3 (see Section S21 in the Supporting Information). At 1000 cd m^−2^, OLED 1 shows an EQE above 15%, exhibiting good resistance to roll‐off with maximum brightness values up to 10 000 cd m^−2^ (EQE = 4.4%). By increasing the concentration of **TAT‐3DBTO_2_** molecules in the emissive layer (OLED 2), the maximum EQE value drops to 20.2% (74 cd m^−2^), but significantly lower efficiency roll‐off is observed. At 10 000 cd m^−2^, OLED 2 shows an EQE of 8.8%, with brightness levels reaching 18 410 cd m^−2^ (EQE = 3.9%).

Figure 6c shows the current density versus voltage. Both OLEDs show very low turn‐on voltages of ≈2.5 V. However, in OLED 1, this value is slightly lower, which may be associated with the fact that **TAT‐3DBTO_2_** molecules are not as ambipolar as BCPO, so by decreasing its concentration, a better *J*–*V* curve (lower turn‐on voltage) is observed. The same explanation holds for the current efficiency (η_c_) versus voltage curves (Figure 6d) up to 8 V: both the devices show similar current efficiency (η_c1,max_ = 50.8 cd A^−1^, η_c2,max_ = 52.9 cd A^−1^), although at higher voltages OLED 1 exhibits much better resistance to high current efficiency levels. **Table** **2** highlights all the electrical properties of these devices and the values of each efficiency at 100 and 1000 cd m^−2^, showing their electrical stability.

**Table 2:** Electrical properties of OLED 1 and OLED 2. *V*_on_ = Turn on voltage; η_ext_ = External quantum efficiency; Brt = brightness; η_c_ = Current efficiency; and η_P_ = Power efficiency. Subscript 100 and 1000 refers to values taken at 100 and 1000 cd m^−2^, respectively

Device	*V*_on_	η_ext,max_ [%]	Brt_max_ [cd m^−2^]	η_c,max_ [cd A^−1^]	η_P,max_ [lm W^−1^]	η_ext,100_ [%]	η_c,100_ [cd A^−1^]	η_P,100_ [lm W^−1^]	η_ext,1000_ [%]	η_c,1000_ [cd A^−1^]	η_P,1000_ [lm W^−1^]
OLED 1	2.3	30.9a)	10 420	50.8	38.7	29	49.8	38.0	16.5	42.9	21.8
OLED 2	2.5	20.2b)	18 410	52.9	44.1	19	49.5	43.8	16.6	49.1	32.4

^a)^ At 76 cd m^−2^

^b)^ At 74 cd m^−2^.

The reproducibility of these devices with such high EQEs and low roll‐offs was studied in more detail (see Section S22 in the Supporting Information). Several other sets of devices with slightly distinct device structures also show EQE values around 30%. Therefore, the data presented in the main paper are the most representative among the OLEDs tested.

## 3 Conclusion

In conclusion, **TAT‐3DBTO_2_** introduces a new design for TADF emitters. The multi‐acceptor single‐donor motif imparts a large number of energy states which gives a short prompt ^1^CT lifetime and unitary PLQY. Moreover, we find 12 singlet–triplet state (pairs) within 0.2 eV of each other, which we believe gives rise to a DF component with a very fast rISC rate, on the order of 1 × 10^7^ s^−1^. This shows that it is possible to achieve both a unitary PLQY and a sub‐microsecond TADF lifetime in the same molecule. The conformational complexity of the molecule, however, gives rise to different rISC rates, as observed in the emission decays. Nevertheless, in devices, these optimal photophysical properties translate into an EQE which exceeds 30% at a useful brightness of 76 cd m^−2^. Thus, this new TADF molecular design opens up a new dimension for achieving truly high performance TADF OLEDs and provides a solution to overcome the main concerns of current TADF molecular designs.

## 4 Experimental Section

Three types of samples were studied in this work: i) **TAT‐3DBTO_2_** solutions (10^−3^–10^−5^ m) in methylcyclohexane, toluene, and dichloromethane (CH_2_Cl_2_) solvents; ii) drop‐casted blend film of **TAT‐3DBTO_2_**:mCP 1:9 molar ratio; and iii) evaporated doped films of **TAT‐3DBTO_2_**:BCPO, 1:9 v/v. All the solutions were stirred for several hours to ensure complete dissolution. The films were dispersed onto quartz substrates.

Steady state absorption and emission spectra were acquired using a UV‐3600 Shimadzu spectrophotometer and a Jobin Yvon Horiba Fluoromax 3, respectively. Time‐resolved spectra were obtained by exciting the sample with a Nd:yittrium aluminium garnet (YAG) laser (EKSPLA), 10 Hz, 355 nm or by using a nitrogen laser, 10 Hz, 337 nm. Sample emission was directed onto a spectrograph and gated intensified charged couple device (iCCD) camera (Stanford Computer Optics).

OLED devices were fabricated using precleaned ITO–coated glass substrates purchased from Ossila with a sheet resistance of 20 Ω cm^−2^ and ITO thickness of 100 nm. The OLED devices had a pixel size of 4 mm × 2 mm or 4 mm × 4 mm. The small molecule and cathode layers were thermally evaporated using the Kurt J. Lesker Spectros II deposition chamber at 10^−6^ mbar. All commercial organic compounds were previously purified by vacuum sublimation.

Structures were optimized with the Q‐Chem quantum chemistry package^16^ using the density functional theory (DFT) method with Pople's 6‐31G(d) basis set^17^ and the Perdew–Burke–Ernzerhof's PBE0 functional.^18^ Electronic structure calculations were performed using the time‐dependent density functional theory (TD‐DFT) method with the 6‐31G(d) basis set and the PBE0 functional corrected by the Tamm–Dancoff approximation.^19^ All calculations were performed in the gas phase.

## Conflict of Interest

The authors declare no conflict of interest.

## Supporting information

Supplementary
