# Supplementary material for: Triazatruxene: A Rigid Central Donor Unit for a D–A3 Thermally Activated Delayed Fluorescence Material Exhibiting Sub‐Microsecond Reverse Intersystem Crossing and Unity Quantum Yield via Multiple Singlet–Triplet State Pairs
Source: Adv Sci (Weinh). 2018 Apr 16;5(6):1700989. doi: 10.1002/advs.201700989 (PMC6010696; doi:10.1002/advs.201700989)
Supplement: Supplementary file 1 — Supplementary [file ADVS-5-1700989-s001.pdf]

Copyright WILEY-VCH Verlag GmbH & Co. KGaA, 69469 Weinheim, Germany, 2018.

ADVANCED SCIENCE
Open Access

Supporting Information

for *Adv. Sci.,* DOI: 10.1002/advs.201700989

Triazatruxene: A Rigid Central Donor Unit for a D–A_3 Thermally Activated Delayed Fluorescence Material Exhibiting Sub-Microsecond Reverse Intersystem Crossing and Unity Quantum Yield via Multiple Singlet–Triplet State Pairs

Paloma L. dos Santos, Jonathan S. Ward, Daniel G. Congrave, Andrei S. Batsanov, Julien Eng, Jessica E. Stacey, Thomas J. Penfold, Andrew P. Monkman, and Martin R. Bryce**

Supporting Information

Triazatruxene: A rigid central donor unit for a D-A_3 thermally activated delayed fluorescence material exhibiting sub-microsecond reverse intersystem crossing and unity quantum yield *via* multiple singlet-triplet state pairs

Paloma L. dos Santos†, Jonathan S. Ward†, Daniel G. Congrave, Andrei S. Batsanov, Julien Eng, Jessica E. Stacey, Thomas J. Penfold, Andrew P. Monkman, Martin R. Bryce**

†These authors contributed equally to this manuscript

S1: General chemistry experimental details

All reactions were carried out under an argon atmosphere unless otherwise stated. Starting materials were purchased commercially and were used as received. Solvents were dried using an Innovative Technology solvent purification system and were stored in ampoules under argon.

TLC analysis was carried out using Merck Silica gel 60 F_{254} TLC plates and spots were visualised using a TLC lamp emitting at 365, 312 or 254 nm. Silica gel column chromatography was performed using silica gel 60 purchased from Sigma Aldrich.

^{1}H and ^{13}C NMR spectroscopy was carried out on Bruker AV400, Varian VNMRS 600 and 700, and Varian Inova 500 NMR spectrometers. Residual solvent peaks were referenced as described in the literature[1], and all NMR data was processed in MestReNova V11.

Melting points were carried out on a Stuart SMP40 machine with a ramping rate of 4 °C min^{-1}. Videos were replayed manually to determine the melting point.

TGA analysis was carried out on a Perkin Elmer Pyris 1 machine with helium gas at 30 mL min^{-1}. Measurements were carried out from 25 °C – 600 °C at 10 °C min^{-1}.

High resolution mass spectrometry was carried out on a Waters LCT Premier XE using ASAP ionisation. Samples were analyzed directly as solids.

Triazatruxene was synthesized using a literature procedure.[2] BCPO was synthesized using a literature procedure.[3]

For the oxidation processes cyclic voltammetry experiments were recorded using a BAS CV50W electrochemical analyzer fitted with a three-electrode system consisting of a glassy carbon (ϕ= 3 mm) working electrode, and Pt wire counter and quasi reference electrodes. Cyclic voltammetry experiments were conducted at a scan rate of 100 mV s^{-1}. Experiments were conducted in dry, deoxygenated CH_2Cl_2 solution with *n*-Bu_4NPF_6 (0.1 M) as the supporting electrolyte. Differential pulse voltammetry experiments were recorded using a PalmSens EmStat[2] potentiostat with PSTrace software. Experiments were conducted with step potentials (E_{step}), pulse potentials (E_{pulse}), and pulse times (t_{pulse}) of 5 mV, 100 mV, and 0.1 s, respectively, at a scan rate of 10 mV/s. All experiments were referenced internally to ferrocene.

For the reduction processes, cyclic voltammetry experiments were recorded using a BioLogic potentiostat SP-300. The electrochemical cell comprised of a Pt disk working electrode, a Ag/AgCl quasi reference electrode and a Pt wire counter electrode. Cyclic voltammetry experiments were conducted at a scan rate of 50 mV s^{-1}. Experiments were conducted in dry, deoxygenated, tetrahydrofuran solution with *n*-Bu_4NBF_4 (0.1 M) as the supporting electrolyte. All experiments were referenced internally to ferrocene.

Redox processes are assigned as being electrochemically reversible based on the equal magnitudes of corresponding oxidation and reduction peaks.

X-ray diffraction experiment was carried out on a Bruker 3-circle D8 Venture diffractometer with a PHOTON 100 CMOS area detector, using Mo-K_α radiation (λ=0.71073Å) from a IμS microsource with focusing mirrors. The crystal was cooled to 120 K using a Cryostream (Oxford Cryosystems) open-flow N_2 gas cryostat. *Crystal data*: $C_{60}H_{33}N_3O_6S_3{\cdot}2CH_2Cl_2$ (*M*=1157.92), monoclinic, space group $P\bar{1}$ (no. 2), *a*= 12.6744(9), *b*= 14.769(1), *c*= 16.671(1)

Å, α = 64.713(2), β = 74.518(3), γ = 66.050(2)°, V= 2562.1(5) $Å^3$, Z= 2, μ(Mo-$K\alpha$)= 0.41 mm^{-1}, D_c= 1.501 g/cm^3, 43714 reflections measured (2θ ≤ 52°), of which 10075 unique (R_{int}= 0.042). The structure was solved by direct methods using SHELXS 2013/1 software[4] and refined by full-matrix least squares using SHELXL 2016/6[5] and OLEX2[6] software, converging to R_1 = 0.069 for 7674 reflections with $I > 2\sigma(I)$ and wR_2= 0.195 on all data. Full crystallographic data has been deposited with the Cambridge Crystallographic Data Centre, dep. no. CCDC-1563363.

S2: Synthesis of TAT-3DBTO$_2$

2-bromodibenzothiophene-*S*,*S*-dioxide (2.87 g, 9.72 mmol, 3.36 eq.), and triazatruxene (1.00 g, 2.89 mmol, 1.00 eq.) were dried under vacuum for one hour in a two-neck 500 mL round-bottomed flask fitted with a reflux condenser. The flask was back-filled with argon and dry toluene (250 mL) was added. The reaction mixture was bubbled with argon for 30 minutes, then $Pd_2(dba)_3 \cdot CHCl_3$ (300 mg, 289 μmol, 0.1 eq.) and $HP^tBu_3BF_4$ (160 mg, 551 μmol, 0.19 eq.) were added and the reaction mixture was bubbled with argon for a further 30 minutes. NaO^tBu (1.31 g, 13.6 mmol, 4.7 eq.) was added under a high flow of argon and the reaction was then heated to 110 °C with stirring for 18 h. The reaction mixture was cooled to room temperature and water (100 mL) was added. The mixture was extracted with EtOAc (3 × 300 mL). The organic layers were combined and washed with water (200 mL) and brine (200 mL). The organic layer was dried with $MgSO_4$ and was filtered. Removal of solvent under reduced pressure gave crude product. The crude mixture was purified by silica gel column chromatography passing through CH_2Cl_2 (2 L) to remove minor fluorescent impurities followed by 1% acetone: CH_2Cl_2 (*v/v*) to elute the product. Removal of solvent under reduced

pressure gave yellow product. The product was recrystallized by stirring in boiling ethanol (200 mL) and slowly adding acetone (≈ 300 mL) until dissolution was almost achieved. The mixture was hot filtered through a glass sinter (grade 4) and ¾ of the filtrate solvent was removed under reduced pressure carefully to ensure no precipitation occurred. Cooling down to −18 °C resulted in product precipitation. The product was filtered and washed with n-pentane (into the filtrate) to give the pure title product as a yellow solid. Further cooling of the filtrate resulted in further crops of pure material (total mass of product 1.15 g, 40% yield). **TAT-3DBTO$_2$** cannot be purified by bulk sublimation on a large scale; however the molecule is volatile enough to be used for evaporated OLED devices due a thin layer being required. **TAT-3DBTO$_2$** was purified by heating at 370 °C for 72 hours at < 5.00 × 10^{-6} mbar to sublime away very minor low molecular weight impurities. The pure material remaining in the sublimation crucible was then used for OLED devices as the TGA analysis (see SI3), at 10 °C min^{-1} ramp rate under He gas, shows stability of the molecule at 370 °C in the absence of oxygen. ^{1}H NMR analysis also shows pure material following the heat treatment under vacuum.

^{1}H NMR (500 MHz, DMSO-d$_6$, 353 K) δ 8.63 (s, 3H), 8.26 (d, *J* = 8.1 Hz, 3H), 8.19 (d, *J* = 7.6 Hz, 3H), 8.04 (d, *J* = 7.7 Hz, 3H), 7.91 (d, *J* = 8.1 Hz, 3H), 7.77 (t, *J* = 7.6 Hz, 3H), 7.72 (t, *J* = 7.6 Hz, 3H), 7.57 (d, *J* = 8.2 Hz, 3H), 7.27 (t, *J* = 7.7 Hz, 3H), 6.82 (t, *J* = 7.7 Hz, 3H), 6.39 (d, *J* = 8.1 Hz, 3H).^{1}H NMR (400 MHz, DMSO-d$_6$, 296 K) δ 8.76 (m, 3H), 8.29 (m, 6H), 8.10 (apr. dd, *J* = 7.5, 2.9 Hz, 3H), 7.82 (m, 9H), 7.60 (apr. d, *J* = 8.2 Hz, 3H), 7.28 (t, *J* = 7.7 Hz, 3H), 6.81 (t, *J* = 7.7 Hz, 3H), 6.31 (apr.d, *J* = 8.2 Hz, 3H); ^{13}C NMR (151 MHz, DMSO-d$_6$) δ 145.2, 140.9, 137.6, 136.6, 134.7, 133.5, 131.7, 130.4, 130.3, 130.1, 124.5, 124.1, 123.6 (br.), 122.74, 122.66, 122.55, 122.2, 121.8, 121.7, 120.7, 110.7, 105.0; HRMS-ASAP^{+}*m/z* calculated for $C_{60}H_{34}N_3O_6S_3$ $[M+H]^{+}$ 988.1579, found: 988.1610; m.p. decomp. > 370 °C under air. TGA: onset 536 °C, mass loss 1.7% (under He).

S3 – TGA analysis for TAT-3DBTO$_2$ under helium gas at 10 °C min^{-1}

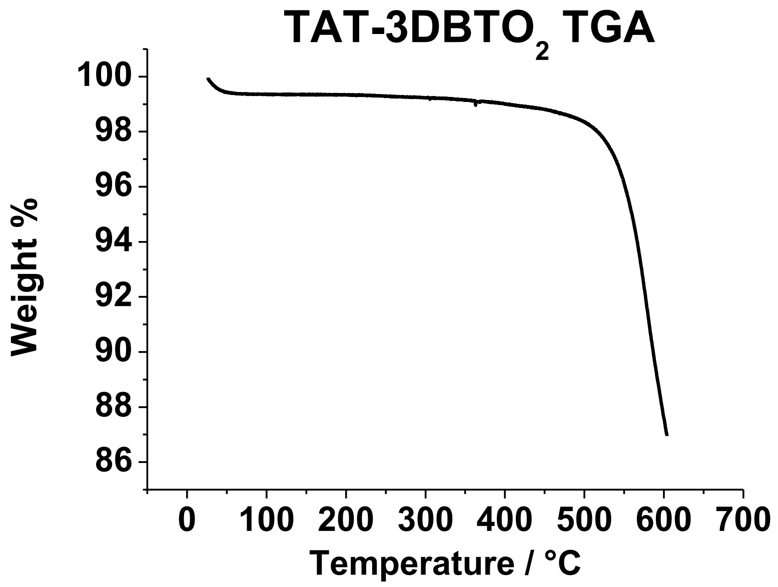

S4: The structure of Triazatruxene (TAT) with position-numbering

Triazatruxene has previously been functionalized in the 2, 7, and 12-positions in many different ways. However, little structural diversity in *N*-substitution has been explored. The vast majority of *N*-functionalization is alkyl-derived to improve solubility with little *N*-aryl functionalization reported.[7] **TAT-3DBTO$_2$** was functionalized on the *N*-position as bonding in this position has typically been shown to give the desirable orthogonal donor and acceptor by twist around the C–N bond between donor and acceptor.

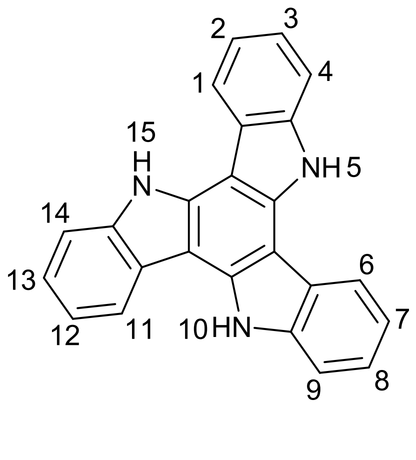

S5: Standard NMR spectra for TAT-3DBTO$_2$

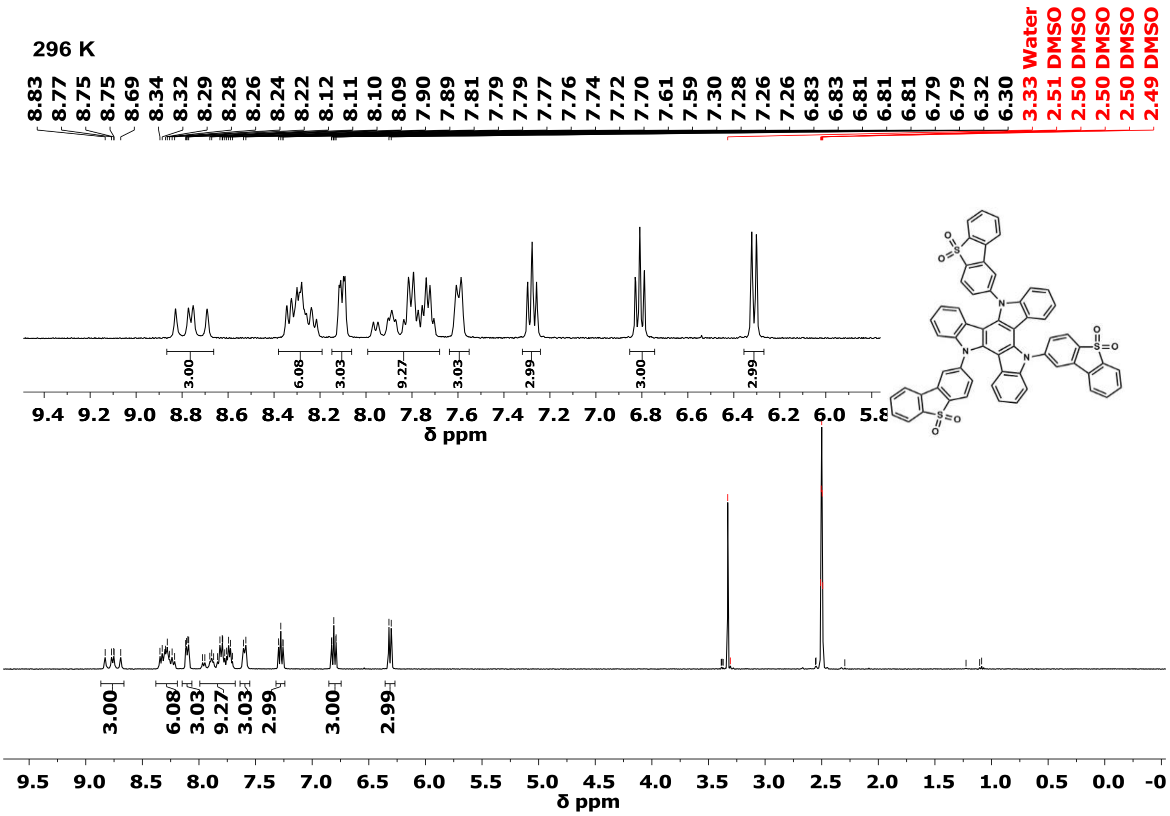

S6: Pure Shift ^{1}H NMR spectrum for TAT-3DBTO$_2$

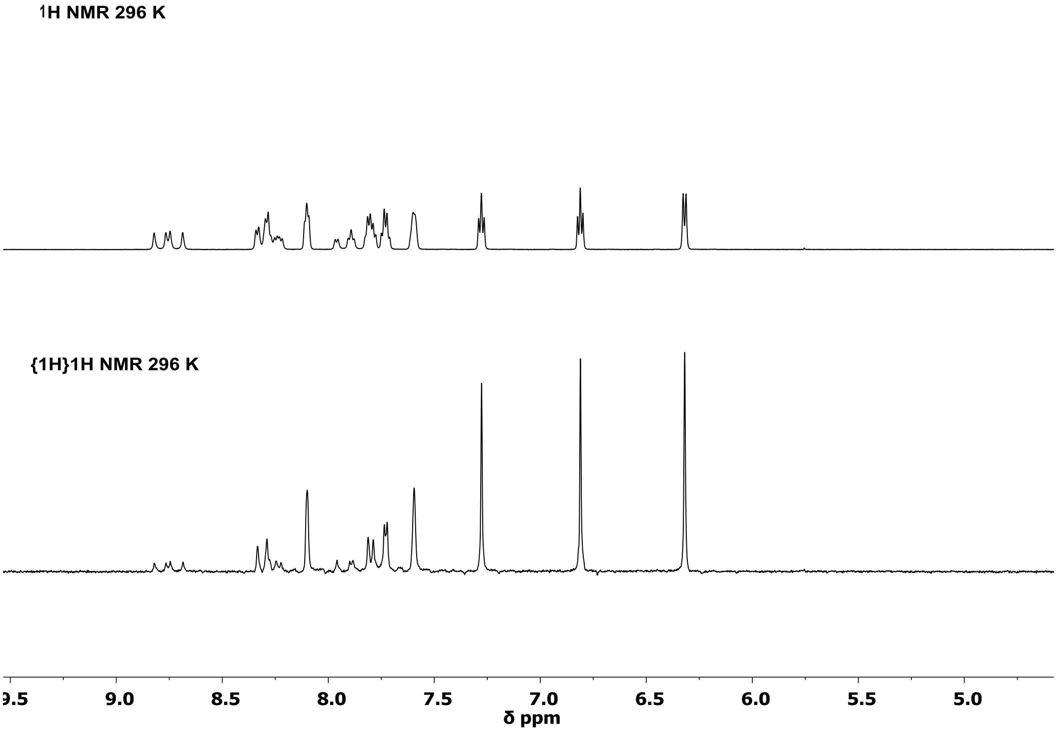

S7: ^{13}C NMR spectrum for TAT-3DBTO$_2$

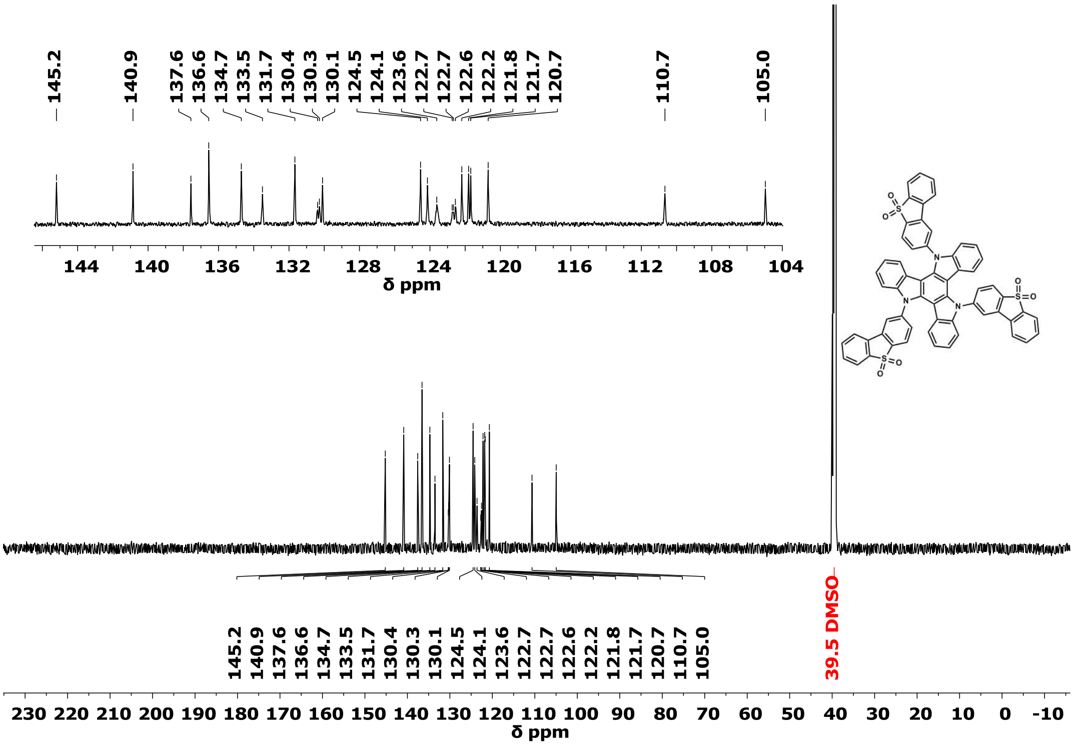

S8: ^{1}H COSY spectrum at 353 K for TAT-3DBTO$_2$

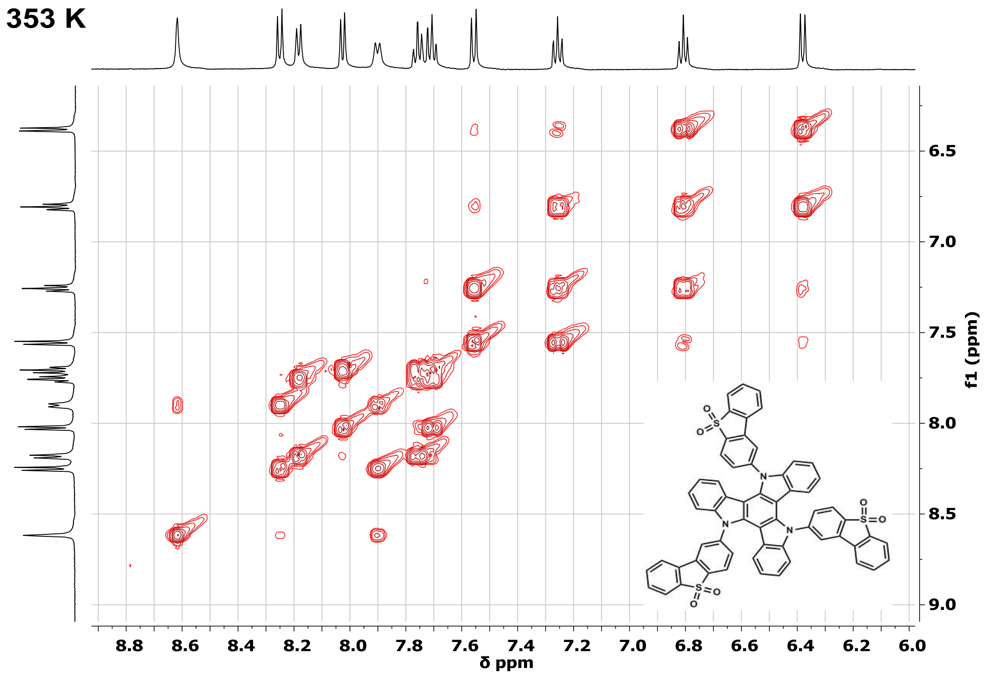

S9: ^{1}H ROESY NMR spectrum at 298 K for TAT-3DBTO$_2$ showing conformer exchange in DMSO-d$_6$ solution, and overcoming barrier to conformer conversion by increasing temperature.

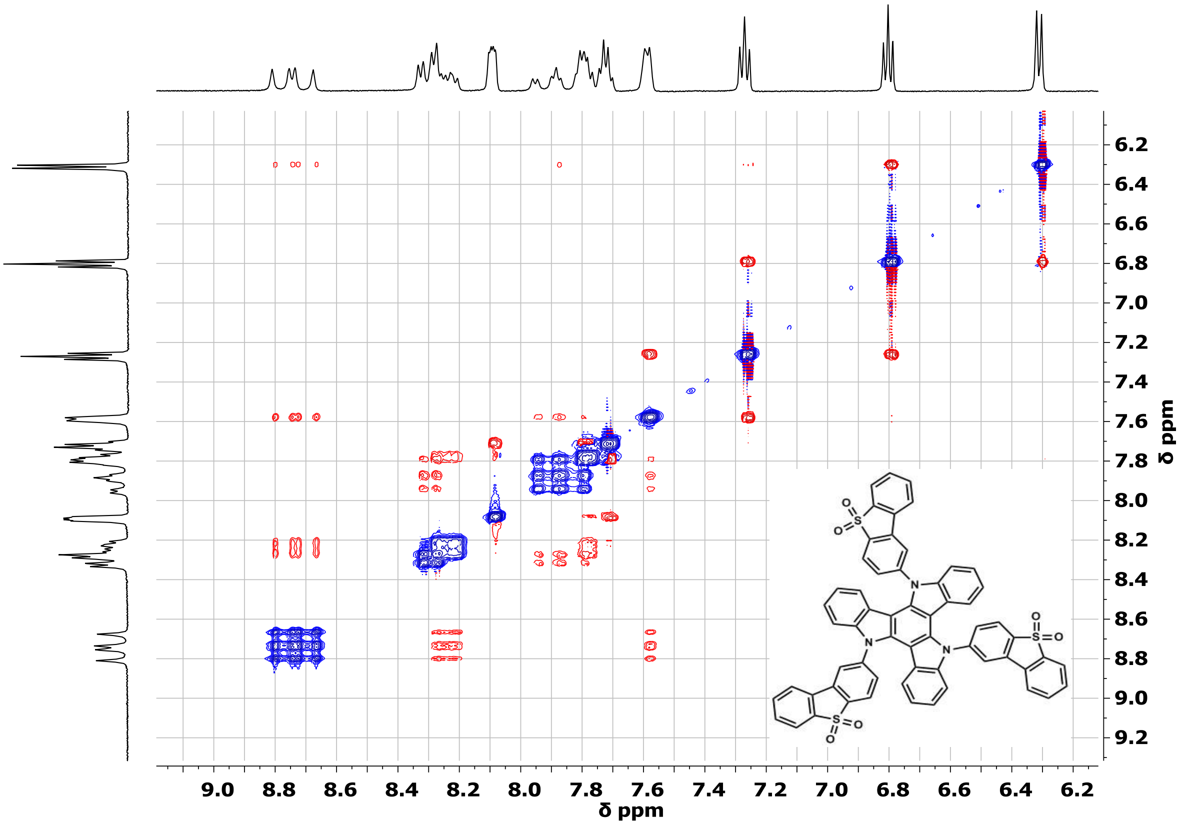

S10: Cyclic and differential pulse voltammograms of TAT-3DBTO$_2$

Cyclic and differential pulse voltammograms showing the oxidation (0.1 M *n*-Bu_4NPF_6/ DCM) and reduction (0.1 M *n*-Bu_4NBF_4/ THF) processes for **TAT-3DBTO$_2$**. Anodic current is defined as positive.

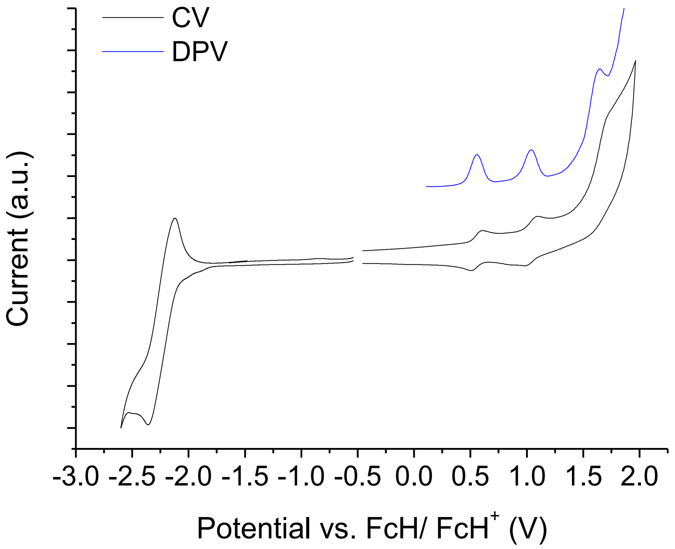

The table below shows the oxidation and reduction potentials ($E_{1/2}^{ox/red}$/ V) of **TAT-3DBTO$_2$** referenced to FcH/ FcH$^+$ = 0.00 V.

$E_{1/2}^{red}$ [onset] / V	$E_{1/2}^{ox(1)}$ [onset] / V	$E_{1/2}^{ox(2)}$/ V	$E_{1/2}^{ox(3)}$/ V	HOMO/ eV^a	LUMO/ eV^b
−2.24 [2.10]	0.56 [0.50]	1.04	1.60	−5.60	−3.00

^{a} HOMO energy calculated from CV potential by HOMO = –5.1 + (–$E_{onset}^{ox(1)}$), using ferrocene as the standard. ^{b} LUMO energy calculated from CV potential by LUMO = –5.1 +(–E_{onset}^{red}), using ferrocene as the standard.[8]

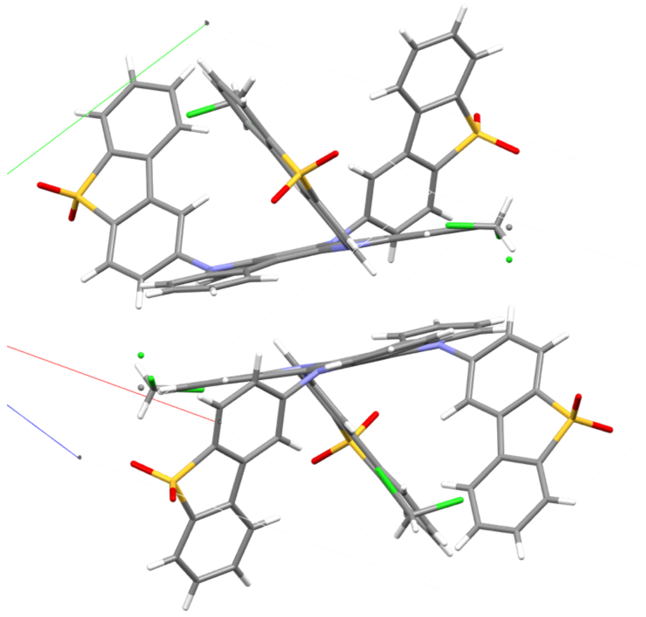

S11: Analysis of the X-ray crystal structure of TAT-3DBTO$_2$

TAT-3DBTO$_2$ has no crystallographic symmetry but an approximate local threefold axis. The central fused donor unit is slightly puckered, the outer arene rings *i*, *ii* and *iii* tilted to one side of the central ring (*iv*) plane by 6.7, 4.0 and 7.0°, respectively. (The ring notation is shown in Figure 2 in the manuscript). The three exocyclic N−C bonds are tilted to the opposite side of plane *iv* by 27.0, 26.1 and 23.4°, the three (planar) dibenzothiophene-*S*,*S*-dioxide are located on the same side and inclined in a propeller-like fashion, forming dihedral angles of 57.6, 61.9 and 59.8° with plane *iv*. The resulting intramolecular cavity is occupied by a CH_2Cl_2 molecule, while another (disordered) CH_2Cl_2 molecule fills an inter-host void. Central moieties of inversion-related molecules are stacked face-to-face, at mean separation of *ca*. 3.6 Å.

From the crystal packing it is seen that the reason that all peripheral acceptor groups appear in the same hemisphere is due to the packing arrangement in the crystal. We believe that this constraint will be relaxed in co-evaporated amorphous films and many of the possible conformers identified in the quantum chemistry calculations will be found in the amorphous films.

S12: Naming convention of different possible conformations of the TAT-3DBTO$_2$

In order to easily name each conformer, we introduce a set of two dimensions for describing the orientation of each acceptor (i) relative to the donor: τ_i is the pyramidalization of the nitrogen atom linked to acceptor i and φ_i is the rotation of the acceptor around the C-N bond. Acceptor units can be either oriented "upwards", $0^o < \varphi_i < 180^o$, or "downwards", $180^o < \varphi_i < 360^o$, with respect to the donor plane. The "upwards" orientation is defined as the direction that most of the acceptor units are oriented. This direction defines the z axis and determines the sign of the angles. If we consider all the acceptor units as equivalent, then we can identify two groups of conformers: the *syn* group, in which all acceptors are oriented in the same direction, and the *anti* group, where one acceptor is oriented in the opposite direction to the two others.

In addition to the orientation, the pyramidalization of the nitrogen atom exhibits two potential wells. The first one with a pyramidalization of $\tau_i < 180^o$, resulting in a *closed* conformation (c), and one with a pyramidalization of $\tau_i > 180^o$ leading to an *open* conformation (o).

S13: Quantum Chemistry studies

Lowest singlet and triplet excited state energies at the minimum of the S_0 geometry for each of the 10 conformers.

ccc-s				cco-s			
**State**	**ΔE / eV**	**ΔE / nm**	**Osc. Str.**	**State**	**ΔE / eV**	**ΔE / nm**	**Osc. Str.**
S0	0.00			S0	0.00		
T1	2.85	435	-	T1	2.85	435	-
T2	2.91	426	-	T2	2.90	427	-
T3	2.91	426	-	T3	2.93	423	-
T4	3.00	413	-	T4	3.01	412	-
S1	3.04	408	0.001	S1	3.03	410	0.020

T5	3.05	406	-	T5	3.04	408	0.031
T6	3.05	406	-	T6	3.04	408	-
S2	3.05	406	0.054	S2	3.06	406	0.025
S3	3.05	406	0.054	S3	3.06	405	-
S4	3.07	404	0.003	S4	3.07	404	0.040
S5	3.10	400	0.015	S5	3.08	402	0.014
S6	3.10	400	0.015	S6	3.10	400	0.012
T7	3.17	391	-	T7	3.16	392	-

**coo-s**				**ooo-s**			
**State**	**ΔE / eV**	**ΔE / nm**	**Osc. Str.**	**State**	**ΔE / eV**	**ΔE / nm**	**Osc. Str.**
S0	0.00			S0	0.00		
T1	2.87	431	-	T1	2.88	431	-
T2	2.92	425	-	T2	2.92	425	-
T3	2.94	422	-	T3	2.92	425	-
T4	3.03	409	-	T4	3.02	411	-
S1	3.04	407	0.028	T5	3.03	409	-
T5	3.04	407	-	T6	3.04	408	-
T6	3.06	406	0.023	S1	3.04	407	0.058
S2	3.07	404	-	S2	3.04	407	0.057
S3	3.07	403	0.061	S3	3.05	406	0.007
S4	3.09	401	0.004	S4	3.07	403	0.005
S5	3.10	400	0.020	S5	3.08	402	0.023

S6	3.12	398	0.019	S6	3.09	402	0.023
T7	3.16	393	-	T7	3.14	394	-

**ccc-a**				**cco-a**			
**State**	**ΔE / eV**	**ΔE / nm**	**Osc. Str.**	**State**	**ΔE / eV**	**ΔE / nm**	**Osc. Str.**
S0	0.00			S0	0.00		
T1	2.86	433	-	T1	2.86	433	-
T2	2.92	425	-	T2	2.91	426	-
T3	2.94	421	-	T3	2.91	426	-
T4	3.02	410	-	T4	3.00	413	-
S1	3.05	406	0.011	S1	3.03	409	0.034
T5	3.06	405	0.034	T5	3.03	409	-
T6	3.07	404	-	T6	3.04	408	0.025
S2	3.07	404	0.047	S2	3.05	406	-
S3	3.09	401	0.022	S3	3.06	406	0.049
S4	3.09	401	-	S4	3.06	405	0.007
S5	3.12	398	0.013	S5	3.09	402	0.011
S6	3.13	396	0.011	S6	3.10	400	0.021
T7	3.18	390	-	T7	3.16	392	-

**coc-a**				**coo-a**			
**State**	**ΔE / eV**	**ΔE / nm**	**Osc. Str.**	**State**	**ΔE / eV**	**ΔE / nm**	**Osc. Str.**
S0	0.00			S0	0.00		

T1	2.87	432	-	T1	2.87	432	-
T2	2.92	424	-	T2	2.92	425	-
T3	2.95	420	-	T3	2.95	420	-
T4	3.02	411	-	T4	3.01	412	-
S1	3.06	406	0.017	S1	3.04	407	0.029
T5	3.07	404	0.042	T5	3.07	404	-
T6	3.07	404	-	T6	3.07	404	0.036
S2	3.08	402	0.012	S2	3.08	403	0.048
S3	3.09	402	0.050	S3	3.08	402	0.011
S4	3.09	401	-	S4	3.09	402	-
S5	3.12	398	0.010	S5	3.11	399	0.015
S6	3.14	395	0.016	S6	3.13	396	0.021
T7	3.16	392	-	T7	3.15	393	-

**ooc-a**				**ooo-a**			
**State**	**ΔE / eV**	**ΔE / nm**	**Osc. Str.**	**State**	**ΔE / eV**	**ΔE / nm**	**Osc. Str.**
S0	0.00			S0	0.00		
T1	2.86	433	-	T1	2.87	432	-
T2	2.91	426	-	T2	2.91	426	-
T3	2.92	425	-	T3	2.93	423	-
T4	3.00	413	-	T4	3.01	412	-
S1	3.03	410	-	S1	3.02	410	-
T5	3.03	409	0.042	T5	3.03	410	0.050
T6	3.05	407	0.031	T6	3.04	408	0.039
S2	3.05	407	-	S2	3.04	408	-

S3	3.05	406	0.047	S3	3.05	407	0.019
S4	3.07	404	0.006	S4	3.06	405	0.015
S5	3.08	403	0.021	S5	3.07	404	0.021
S6	3.10	400	0.020	S6	3.08	403	0.024
T7	3.15	393	-	T7	3.14	394	-

Density difference for the 13 lowest electronic states at S_0 minimum of conformer ccc-s in gas phase at the DFT PBE0/6-31G(d) level of computation. Gain of electronic density in blue: loss in red.

S1/T1

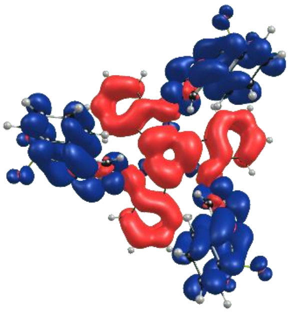

S2/T2

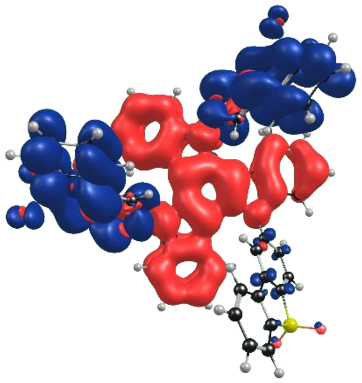

S3/T3

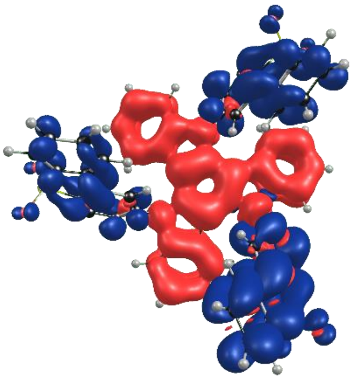

S4/T4

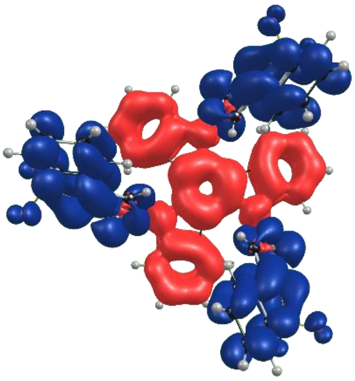

S5/T5

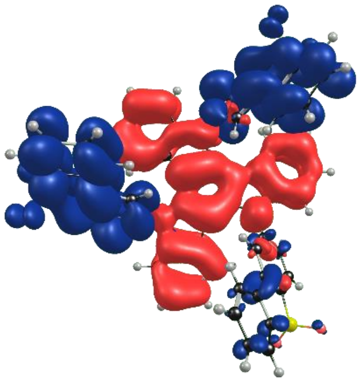

S6/T6

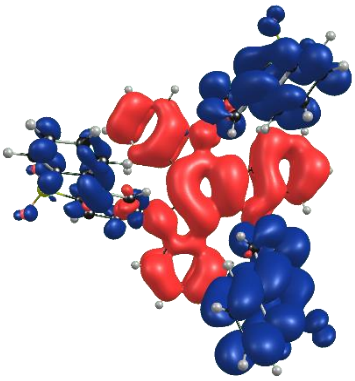

T7

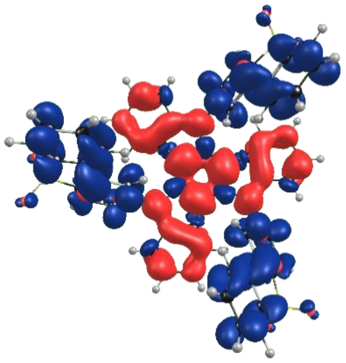

Calculated absorption spectrum for all conformers.

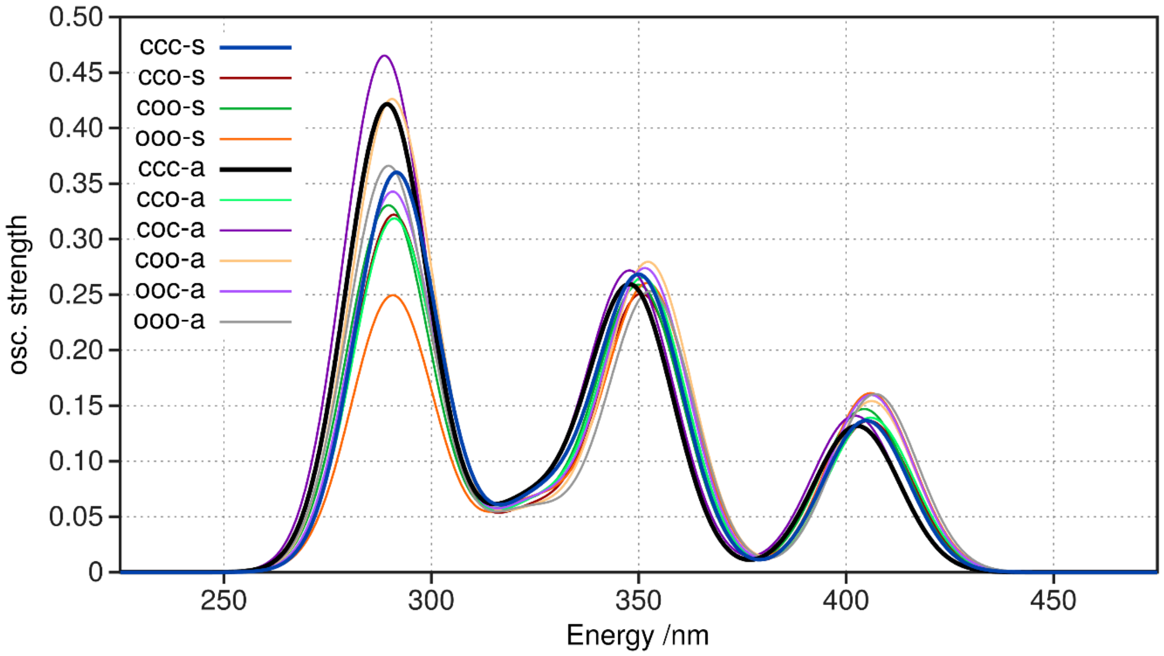

S14: Quantum Chemistry studies

Lowest singlet and triplet excited state energies at the minimum of the S_1 geometry for each of the 10 conformers.

ccp-s				cpo-s			
**State**	**ΔE / eV**	**ΔE / nm**	**Osc. Str.**	**State**	**ΔE / eV**	**ΔE / nm**	**Osc. Str.**
S0	0.00			S0	0.00		

T1	2.13	583	-	T1	2.10	590	-
S1	2.14	**579**	0.001	S1	2.11	**587**	0.001
T2	2.41	515	-	T2	2.36	525	-
S2	2.42	512	0.005	S2	2.37	523	0.003
T3	2.66	467	-	T3	2.67	464	-
T4	2.73	454	-	S3	2.74	452	0.031
S3	2.74	452	0.031	T4	2.75	451	-
T5	2.76	450	-	T5	2.76	449	-
S4	2.77	447	0.000	S4	2.78	445	0.006
T6	2.87	432	-	T6	2.87	433	-
T7	2.91	427	-	T7	2.90	428	-
S5	2.94	422	0.010	S5	2.93	424	0.004
S6	3.01	412	0.029	S6	2.99	414	0.007

**poo-s**				**ccp-a**			
**State**	**ΔE / eV**	**ΔE / nm**	**Osc. Str.**	**State**	**ΔE / eV**	**ΔE / nm**	**Osc. Str.**
S0	0.00			S0	0.00		
T1	2.13	582	-	T1	2.15	577	-
S1	2.15	**578**	0.001	S1	2.16	**574**	0.004
T2	2.40	517	-	T2	2.41	515	-
S2	2.41	515	0.006	S2	2.42	512	0.004
T3	2.67	464	-	T3	2.67	464	-
T4	2.71	458	-	T4	2.72	456	-
S3	2.73	455	0.004	S3	2.75	450	0.016
T5	2.75	450	-	S4	2.76	449	0.013

S4	2.77	448	0.033	T5	2.77	448	-
T6	2.87	433	-	T6	2.87	433	-
T7	2.91	426	-	T7	2.91	426	-
S5	2.94	421	0.030	S5	2.94	422	0.014
S6	2.96	418	0.021	S6	2.99	415	0.022

**cpc-a**				**cpo-a**			
**State**	**ΔE / eV**	**ΔE / nm**	**Osc. Str.**	**State**	**ΔE / eV**	**ΔE / nm**	**Osc. Str.**
S0	0.00			S0	0.00		
T1	2.14	580	-	T1	2.14	581	-
S1	2.15	**578**	0.003	S1	2.15	**576**	0.001
T2	2.40	517	-	T2	2.40	516	-
S2	2.41	515	0.002	S2	2.42	513	0.007
T3	2.67	464	-	T3	2.68	463	-
T4	2.77	448	-	T4	2.70	458	-
T5	2.77	448	-	S3	2.72	455	0.006
S3	2.77	447	0.037	T5	2.75	450	-
S4	2.81	441	0.001	S4	2.80	443	0.042
T6	2.88	431	-	T6	2.87	432	-
T7	2.92	424	-	T7	2.91	426	-
S5	2.97	417	0.009	S5	2.94	421	0.028
S6	2.97	417	0.004	S6	2.99	415	0.012

**cop-a**				**oop-a**			
**State**	**ΔE / eV**	**ΔE / nm**	**Osc. Str.**	**State**	**ΔE / eV**	**ΔE / nm**	**Osc. Str.**

S0	0.00			S0	0.00		
T1	2.13	583	-	T1	2.14	580	-
S1	2.14	**580**	0.002	S1	2.15	**576**	0.001
T2	2.40	517	-	T2	2.41	514	-
S2	2.41	515	0.005	S2	2.42	511	0.005
T3	2.68	462	-	T3	2.67	465	-
T4	2.75	450	-	T4	2.70	458	-
T5	2.75	450	-	S3	2.73	454	0.003
S3	2.78	446	0.034	S4	2.74	453	0.027
S4	2.79	445	0.008	T5	2.76	450	-
T6	2.87	431	-	T6	2.87	432	-
T7	2.93	423	-	T7	2.88	430	-
S5	2.98	415	0.005	S5	2.93	424	0.028
S6	3.02	411	0.047	S6	2.97	417	0.032

**poc-a**				**poo-a**			
**State**	**ΔE / eV**	**ΔE / nm**	**Osc. Str.**	**State**	**ΔE / eV**	**ΔE / nm**	**Osc. Str.**
S0	0.00			S0	0.00		
T1	2.15	576	-	T1	2.14	579	-
S1	2.17	**572**	0.001	S1	2.15	**577**	0.003
T2	2.42	512	-	T2	2.40	517	-
S2	2.43	510	0.006	S2	2.41	515	0.002
T3	2.64	469	-	T3	2.68	462	-
S3	2.72	455	0.025	T4	2.75	452	-

T4	2.73	454	-	S3	2.76	449	0.025
S4	2.76	450	0.002	T5	2.77	448	-
T5	2.76	449	-	S4	2.79	445	0.010
T6	2.88	431	-	T6	2.87	432	-
T7	2.88	430	-	T7	2.92	425	-
S5	2.92	425	0.021	S5	2.95	420	0.019
S6	2.97	417	0.023	S6	3.01	411	0.047

Density difference for the 13 lowest electronic states at S_1 minimum of conformer ccp-s in gas phase at the DFT PBE0/6-31G(d) level of computation. Gain of electronic density in blue: loss in red.

S1/T1

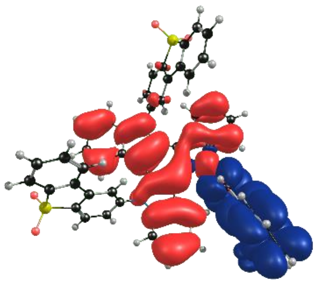

S2/T2

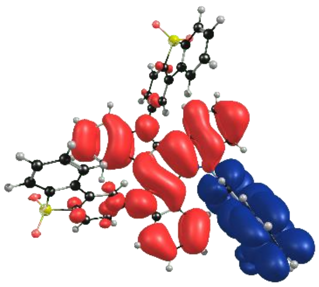

S3/T3

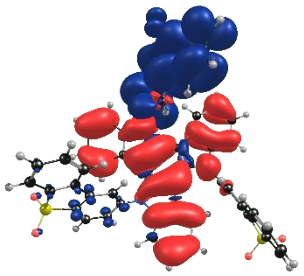

S4/T4

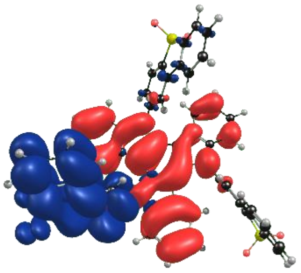

T5

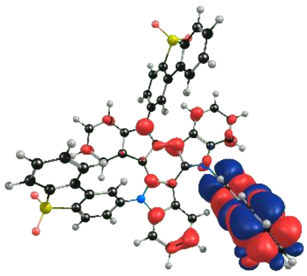

S5/T6

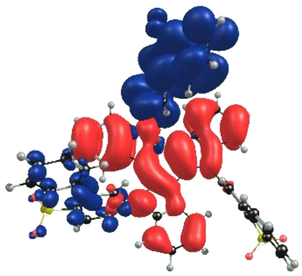

S6/T7

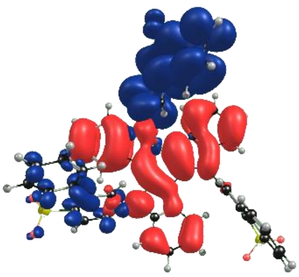

S15: Absorption spectra of TAT-3DBTO$_2$ in different solvents

Figure SI15 shows the normalised absorption spectra of TAT-3DBTO$_2$ in dichloromethane (CH_2Cl_2) and toluene solutions. A slight red shift on the right edge of the spectra is observed by increasing the polarity of the solvent.

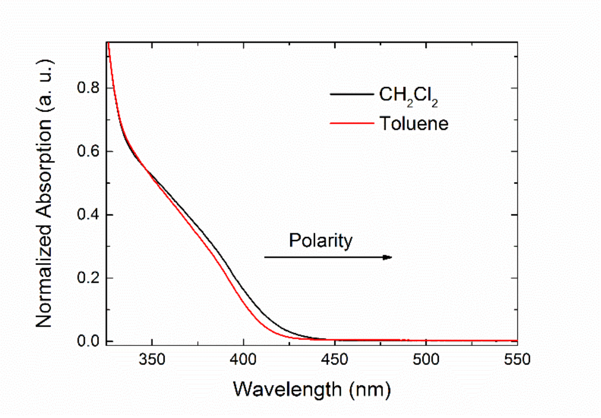

S16: Photoluminescence quantum yields (PLQY) of TAT-3DBTO$_2$:BCPO

The photoluminescence quantum yields (PLQYs) of **TAT-3DBTO$_2$**:BCPO were recorded on a Horiba Jobin Yvon SPEX Fluorolog 3 using a calibrated Quanta-Φ integrating sphere and

were calculated according to the literature method.[9] Solid state PLQY data were obtained in duplicate on two separate films which were prepared in parallel.

The PLQY of the film was evaluated under air at an excitation wavelength of 380 nm and it was found to be $PLQY_{PF}$ = (63 ± 10)%. This value includes mainly the contribution of the prompt fluorescence (PF). To estimate the PLQY with the incorporation of delayed fluorescence (DF), i.e. including the contribution from the triplet states, the PL spectrum of the **TAT-3DBTO$_2$**:BCPO film was collected both under air and vacuum. The red curve refers to the PL spectrum under vacuum and the black curve to the aerated PL. Both PL spectra match each other, showing that DF and PF come from the same ^{1}CT state.

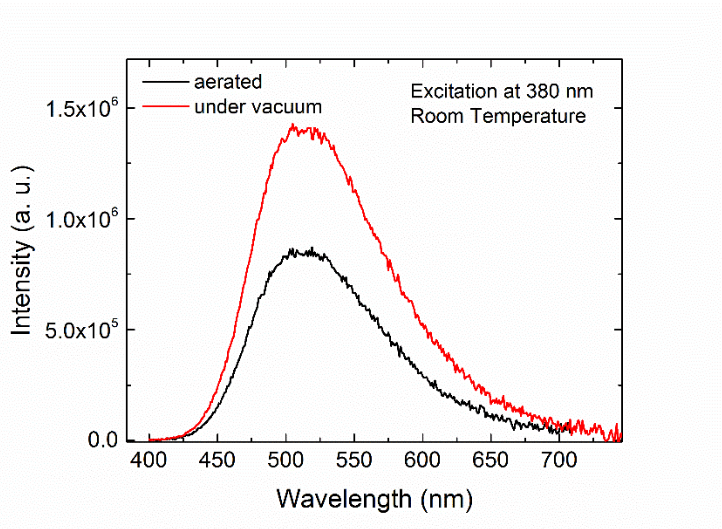

The ratio between the area of the red spectrum ($Area_{PF+DF}$) and the black spectrum ($Area_{PF}$) was found to be 1.69. Thus, the $PLQY_{PF+DF} \approx 100\%$.

$$\frac{Area_{PF+DF}}{Area_{PF}} = \frac{\Phi_{PF+DF}}{\Phi_{PF}} = 1.69$$

The PLQY of neat evaporated **TAT-3DBTO$_2$** films was also measured and found to be 12± 10% in air. This demonstrates that emission quenching occurs in neat **TAT-3DBTO$_2$** films. This is undesirable for studying detailed photophysics and for optimising OLED devices with this molecule. This is why BCPO host was employed in this case. In the figure below , we

give the time resolved emission decay data for a pure TAT-3DBTO2 film at room temperature, having PLQY of 12% in air. As clearly seen, all decay dynamics by rISC are masked by the concentration quenching. This data does not allow us to make a quantitative evaluation of rISC in these films.

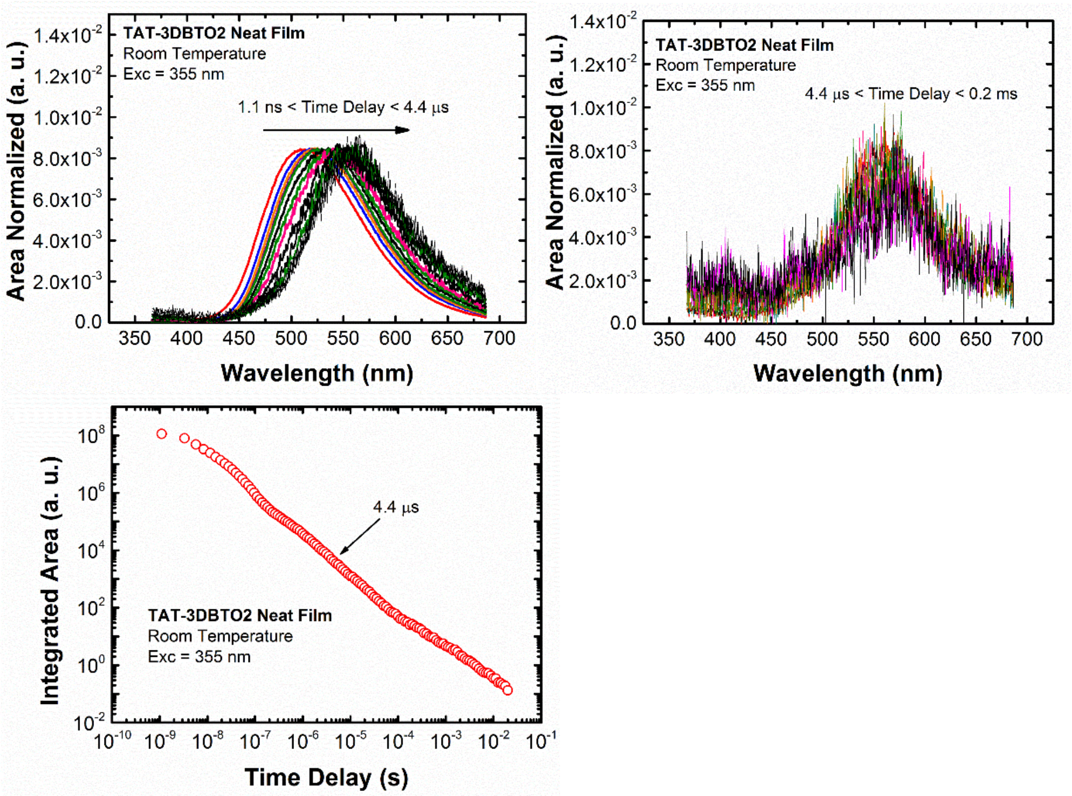

Monotonic red shifting of the ^{1}CT spectrum is observed showing large-scale sample inhomogeneity (in terms of CT-^{3}LE gap), as can be seen in the time resolved emission spectra of TAT-3DBTO2 neat film. These spectra are red-shifted if compared to the TAT-3DBTO2:BCPO emission spectra (Figure 5 manuscript) indicating that TAT-3DBTO2 is more likely more polar than BCPO. Moreover, the decay curve of neat film at room temperature shows PF and DF emission are ill defined. Likely, the TADF mechanism in this neat film is very weak and even TTA may occur. All these characteristics shown by the neat film are not desirable in our study, nor can any quantitative data be extracted from such data, so the full photophysical characterization was only done in TAT-3DBTO2:BCPO film.

S17: Decay curve of TAT-3DBTO$_2$:BCPO film at 320 K

If we compare delayed emission decay, region II in the figure below, at 320 and 280 K, the curves are virtually identical, apart from a longer lifetime tail (phosphorescence component) observed at 280 K (circled), which is expected when decreasing the temperature in efficient TADF systems, see ref 14 a in main paper. Therefore, considering that these two curves have a 40 K temperature step, we expected that at 300 K (half of the step) all the rates will be very similar to the ones calculated at 320 K. As previously shown in many of our previous papers (see ref 1a in main paper), k_{rISC} saturates around 260-280 K Saturation occurs independent of large or small gap.

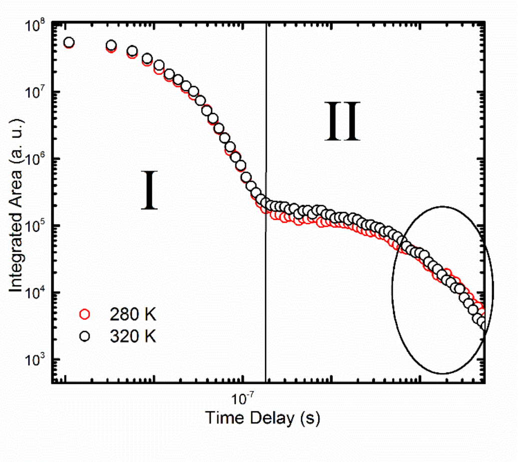

Figure S17 shows the decay curve of **TAT-3DBTO$_2$**:BCPO film at 320 K. The PF decay was fitted with two exponentials and the DF with three exponentials (Curve fitting - OriginPro 9.0). PF was fitted in region I (see manuscript) and DF was fitted in region II (see manuscript), which is the timeframe of strong TADF mechanism.

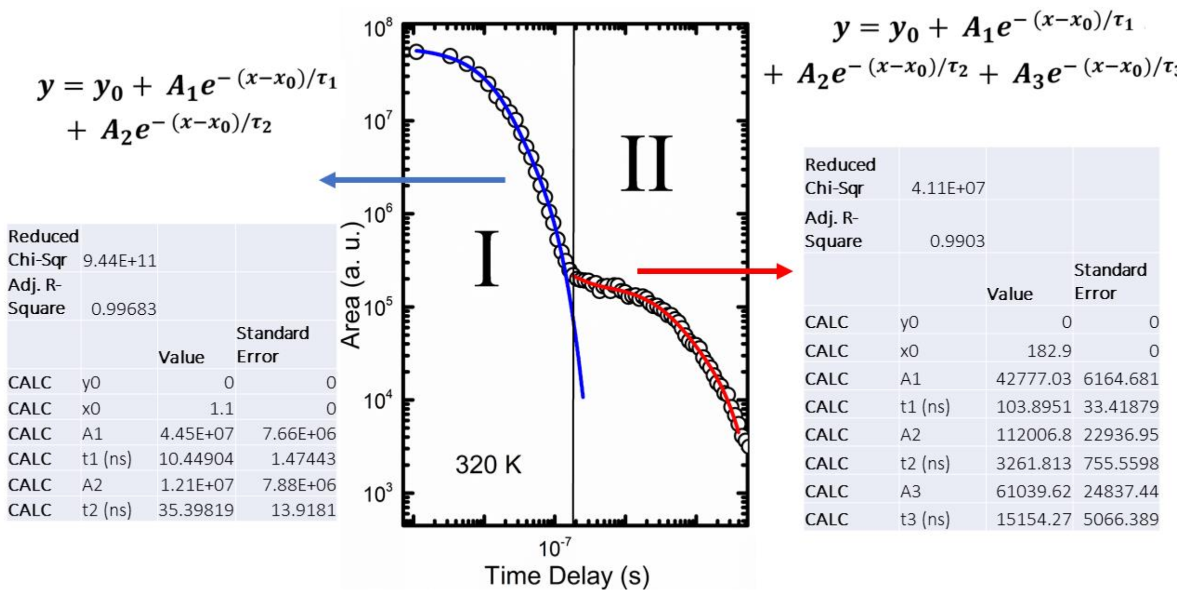

S18: Calculation of k_{rISC} rate

Two different approaches were used to calculate k_{rISC}, equation a and b:

a) $k_{rISC} = \frac{1}{\tau_{DF}} \frac{1}{(1-\Phi_{ISC})}$; b) $k_{rISC} = \frac{\int I_{DF}(t)dt}{\int I_{PF}(t)dt} . \frac{1}{\tau_{DF}}$

Approach *a*, relates the k_{rISC} with the triplet formation yield (Φ_{ISC}), which was measured at 37%, see supporting information SI16. Approach *b*, relates k_{rISC} with the integrated area of the PF and DF emission, $\int I_{PF}(t)dt$ and $\int I_{DF}(t)dt$, respectively, found from the decay curves. The integrated area of DF emission was evaluated in the region where the TADF mechanism is active (region II). All the values are found in the table below:

**Equation 1a**	$k^a_{rISC} = \frac{1}{\tau_{DF}} . \frac{1}{(1-\Phi_{ISC})}$	**Equation 1b**	$k^b_{rISC} = \frac{\int I_{DF}(t)dt}{\int I_{PF}(t)dt} . \frac{1}{\tau_{DF}}$
$\boldsymbol{\Phi_{ISC}}$	0.37	$\boldsymbol{\int I_{PF}(t)dt}$	901724916.9

**1- Φ_{ISC}**	0.63	$\int I_{DF}(t)dt$	1258603656
**τ_1 (s)**	1.04×10^{-7}	**τ_1 (s)**	1.04×10^{-7}
**k_{rISC} (τ_1) (s^{-1})**	1.53×10^{7}	**k_{rISC} (τ_1) (s^{-1})**	1.34×10^{7}
**τ_2 (s)**	3.26×10^{-6}	**τ_2 (s)**	3.26×10^{-6}
**k_{rISC} (τ_2) (s^{-1})**	4.87×10^{5}	**k_{rISC} (τ_2) (s^{-1})**	4.28×10^{5}
**τ_3 (s)**	1.52×10^{-5}	**τ_3 (s)**	1.52×10^{-5}
**k_{rISC} (τ_3) (s^{-1})**	1.05×10^{5}	**k_{rISC} (τ_3) (s^{-1})**	9.21×10^{4}

S19: Excitation dose dependence of TAT-3DBTO$_2$:BCPO film

Figure SI19 shows the area of DF spectra of **TAT-3DBTO$_2$**:BCPO film as a function of the excitation dose. The line of best fit shows a slope of 0.9. This result confirms the thermally activated mechanism as opposed to triplet-triplet annihilation (TTA).

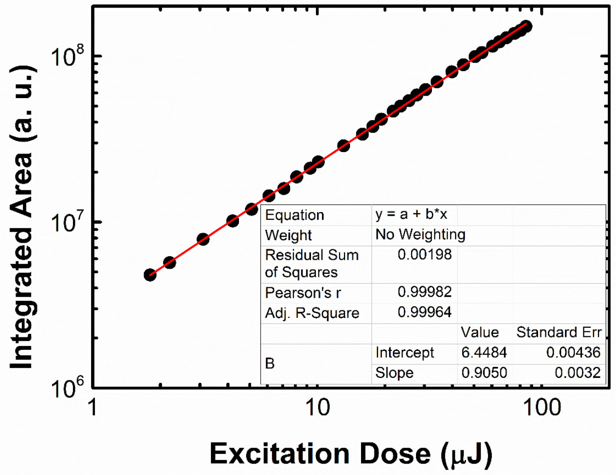

S20: Phosphorescence (PH) spectra of TAT-3DBTO$_2$

Figure S20 shows the PL and PH spectra of **TAT-3DBTO$_2$**:mCP (1:9, molar ratio). The PL spectrum was collected at room temperature and PH at 80 K. The PH was collected with delay time of 70 ms and integration time of 10 ms. Both experiments were performed with excitation at 355 nm. Dash lines show how the onset values were calculated from the spectra.

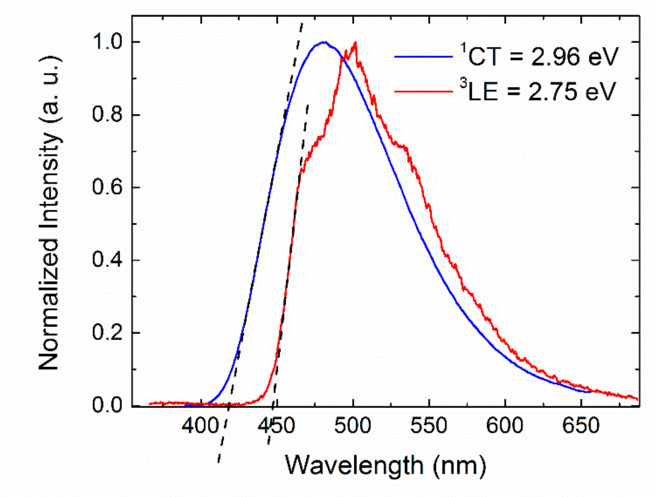

The PH spectrum of **TAT-3DBTO$_2$** was also collected in another environment, polyethylene oxide, to investigate if the change in the environmental polarity would change the onset of the PH spectra. As can be seen below, the PH spectra collected in polyethylene oxide matrix and mCP host show the same onset values.

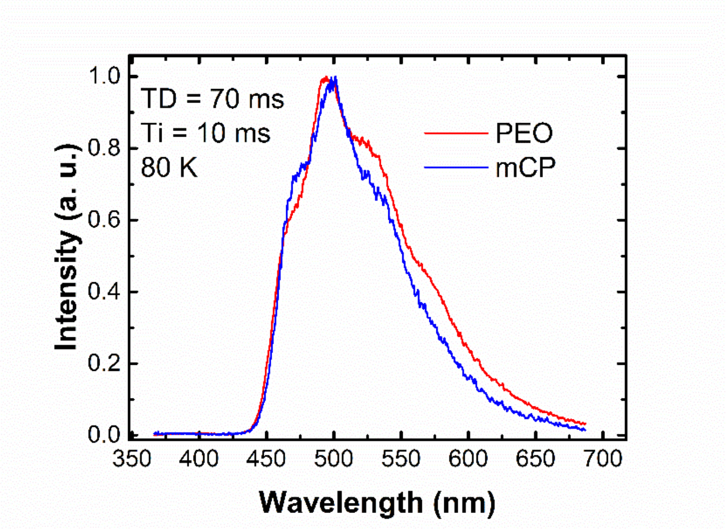

S21: Analyses of EQE values obtained in OLED 1

The EQE of an OLED is described by the equation below as the product between the charge balance factor, γ, the fraction of spin-allowed excitons, $\eta_{ST,}$ the photoluminescence quantum yield, Φ_{PL} and the outcoupling efficiency, $\eta_{out,}$ which is usually estimated 20 - 30%.[10]

$$EQE = \gamma \cdot \eta_{ST} \cdot \Phi_{PL} \cdot \eta_{out},$$

For OLED 1, Φ_{PL} =1 and the high EQE values, we can conclude that the device has a charge balance close to unity (γ =1); all the excitons are harvested from the triplet to the singlet states, i.e., 100% TADF efficiency (η_{ST} =1); under the assumption that η_{out} ~ 0.3.

S22: Optimization of TAT-3DBTO$_2$:BCPO devices

Figure SI22 shows the EQE *versus* brightness of eight devices (four distinct structures). The structures of the devices and EQE values are presented in the table below.

BCPO was found to be the most suitable host for **TAT-3DBTO$_2$** due to many factors: suitable HOMO, LUMO and triplet level, ambipolar properties allowing transport of holes and electrons throughout the entire emissive layer, the small ΔE_{ST} of **TAT-3DBTO$_2$** in this host due to the correct polarity arising from the P=O bond and carbazole moiety, and high photoluminescence quantum yield (PLQY) ≈ 100% measured for **TAT-3DBTO$_2$** in BCPO film.

The device optimization aiming at low roll-off was studied previously (OLED A1, A2 and OLED 2 in the manuscript), and in here, the optimization of maximum EQE values is demonstrated. Intending to increase the EQE values, a much smaller amount of **TAT-3DBTO$_2$** was co-evaporated with BCPO, 4% (OLED B1 and B2). However, together with a strong increase in the maximum EQE values, a big increase in the roll-off was also observed. To retain the high EQE values without significantly sacrificing the roll-off, devices with 10% **TAT-3DBTO$_2$** were produced (OLED C1, C2 and OLED 1 in the manuscript). As the ratio ~ 10% guest:host was observed to be the best for maximum EQE values, devices were also

fabricated with buffer layers of BCPO aiming at more balanced carrier density in the emissive layer. However, no significant increase in the performance was observed.

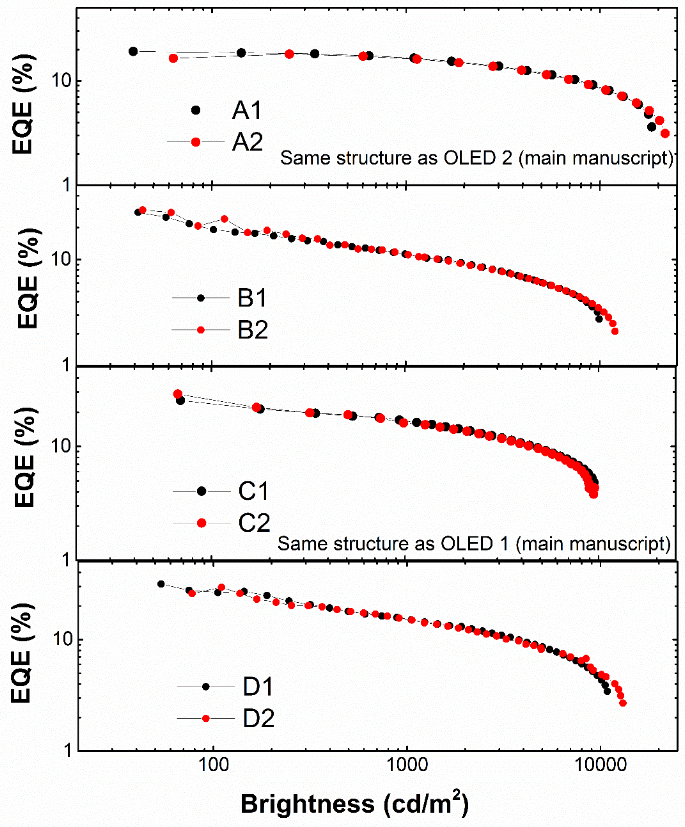

OLED	Hole Transport	Layers	Emissive Layer	Electron Transport Layer	Max EQE	EQE at 1,000 cd/m^2
**A1**	NPB (40 nm)	TCTA (10nm)	TAT-3DBTO$_2$:BCPO (1.7:8.3)	TPBi (50 nm)	19.2% 39 cd/m^2	16.7%
**A2**	NPB (40 nm)	TCTA (10nm)	TAT-3DBTO$_2$:BCPO (1.7:8.3)	TPBi (50 nm)	18.3% 250 cd/m^2	16.4%
**B1**	NPB (40 nm)	TCTA (10nm)	TAT-3DBTO$_2$:BCPO (0.4:9.6)	TPBi (50 nm)	27.9% 42 cd/m^2	11.2%
**B2**	NPB (40 nm)	TCTA (10nm)	TAT-3DBTO$_2$:BCPO (0.4:9.6)	TPBi (50 nm)	29.4% 44 cd/m^2	11.3%
**C1**	NPB (40 nm)	TCTA (10nm)	TAT-3DBTO$_2$:BCPO (1:9)	TPBi (50 nm)	25.4% 69 cd/m^2	16.7%
**C2**	NPB (40 nm)	TCTA (10nm)	TAT-3DBTO$_2$:BCPO (1:9)	TPBi (50 nm)	28.9% 67 cd/m^2	16%
**D1**	NPB (40 nm)	TCTA (10nm)	BCPO(5nm)/ TAT3DBTO$_2$:BCPO (0.8:9.2)/ BCPO (5nm)	TPBi (50 nm)	31.5% 53 cd/m^2	15.2%
**D2**	NPB (40 nm)	TCTA (10nm)	BCPO(5nm)/ TAT3DBTO$_2$:BCPO (0.8:9.2)/ BCPO (5nm)	TPBi (50 nm)	29.4% 110 cd/m^2	15.2%

S23: References

[1] G. R. Fulmer, A. J. M. Miller, N. H. Sherden, H. E. Gottlieb, A. Nudelman, B. M. Stoltz, J. E. Bercaw, K. I. Goldberg, *Organometallics* **2010**, *29*, 2176.

[2] K. Rakstys, A. Abate, M. I. Dar, P. Gao, V. Jankauskas, G. Jacopin, E. Kamarauskas, S. Kazim, S. Ahmad, M. Gratzel, M. K. Nazeeruddin, *J. Am. Chem. Soc.* **2015**, *137*, 16172.

[3] H. H. Chou, C. H. Cheng, *Adv. Mater.* **2010**, *22*, 2468.

[4] G. M. Sheldrick, *Acta Crystallogr. A* **2008**, *64*, 112.

[5] G. M. Sheldrick, *Acta Crystallogr. C Struct. Chem.* **2015**, *71*, 3.

[6] O. V. Dolomanov, L. J. Bourhis, R. J. Gildea, J. A. K. Howard, H. Puschmann, *J. Appl. Crystallogr.* **2009**, *42*, 339.

[7] a) K. Rakstys, S. Paek, P. Gao, P. Gratia, T. Marszalek, G. Grancini, K. T. Cho, K. Genevicius, V. Jankauskas, W. Pisula, M. K. Nazeeruddin, *J. Mater. Chem. A* **2017**, *5*, 7811; b) L. Wang, Q. Fang, Q. Lu, S. J. Zhang, Y. Y. Jin, Z. Q. Liu, *Org. Lett.* **2015**, *17*, 4164.

[8] J. S. Ward, R. S. Nobuyasu, A. S. Batsanov, P. Data, A. P. Monkman, F. B. Dias, M. R. Bryce, *Chem. Commun.* **2016**, *52*, 2612.

[9] L. O. Palsson, A. P. Monkman, *Adv. Mater.* **2002**, *14*, 757.

[10] a) M. C. Gather, S. Reineke, *J. Photon. Energy* **2015**, *5*, 057607; b) N. K. Patel, S. Cina, J. H. Burroughes, *IEEE J. Sel. Top. Quantum Electron.* **2002**, *8*, 346; c) D. Pereira, P. Data, A. Monkman, *Display and Imaging* **2017**, *2*, 323.
